# The impact of high fat diet on global protein abundance and fractional synthetic rate in liver and mammary gland of peak lactation ICR mice

**DOI:** 10.1371/journal.pone.0346148

**Published:** 2026-04-10

**Authors:** Linda Beckett, Nathanael Lichti, Kelsey Teeple, Venkatesh Thirumalaikumar, Laura Hernandez, Bartek Rajwa, Amber Jannasch, Yu Han-Hallett, Theresa Casey

**Affiliations:** 1 Department of Animal Sciences, Purdue University, West Lafayette, Indiana, United States of America; 2 Bindley Bioscience Center, Purdue University, West Lafayette, Indiana, United States of America; 3 Department of Botany and Plant Pathology, Purdue University, West Lafayette, Indiana, United States of America; 4 Department of Animal and Dairy Sciences, University of Wisconsin, Madison, Wisconsin, United States of America; University of South Carolina, UNITED STATES OF AMERICA

## Abstract

Maternal metabolic environment creates the developmental environment for offspring. Previous studies demonstrated high fat (HF) diet increased neonate growth rate during lactation, which related to increased milk lactose content and increased fatty acyl chain length and unsaturation of milk lipids. To understand how HF diet alters maternal metabolism, we measured liver and mammary gland global protein abundance and fractional synthetic rate (FSR) of peak lactation mice after enriching body water with deuterium oxide for 24 h. In both tissues, HF altered abundance of proteins that reflected less dependence on glycolysis and a greater dependence on fatty acid degradation for energy production. Alterations in fatty acid profiles of milk due to HF diet linked to decreased abundance of enzymes that mediate *de novo* fatty acid synthesis and mono-unsaturation, and increased abundance of enzymes that function in the elongation and desaturation of polyunsaturated fatty acids. In the liver, HF diet increased ketogenic and gluconeogenic enzymes, indicating higher production of ketones and glucose, the former potentially linked to reduction in mammalian target of rapamycin (mTOR) abundance and the latter potentially explaining increased milk lactose content. The higher abundance of ribosomal proteins in the mammary gland of HF mice may promote greater milk production capacity and thus partly explain greater growth rate of offspring. Among proteins with significantly different FSR, HF diet decreased FSR of ~82% of the proteins in liver and ~80% in mammary. These findings enhance understanding of the impacts of diets on maternal metabolism and milk production during lactation, and expand the general understanding of how HF diet impacts metabolic pathways and proteostatic processes.

## Introduction

Coordinated changes in maternal metabolism and behavior occur to support the nutrient and energetic demands of the growing fetus during pregnancy and suckling neonate during lactation. Changes include increased feed intake, increased storage and then mobilization of fat and protein, and increased organ sizes including heart, gastrointestinal tract, mammary glands and liver [1–7]. External environment and maternal nutrition impact the dam’s physiological and behavioral adaptations to pregnancy and lactation, and these adaptions create the metabolic environment of the developing offspring. Findings from both human and animal studies show a clear connection between early exposure to maternal high fat (HF) diet during pregnancy and lactation and poorer long-term health of offspring, with maternal HF diet increasing offspring risk for metabolic syndrome, hypertension, type 2 diabetes, obesity, cardiovascular dysfunction and alterations in neurological development [8–13]. Rodent postnatal cross-fostering studies demonstrated that offspring exposed to maternal HF diet only during lactation develop a metabolic-like syndrome as adults [13], indicating that maternal nutritional environment during lactation alone programs offspring development.

To enable milk production at the onset of lactation, the mammary gland increases its capacity for nutrient uptake, energy generation, and protein, fatty acid, and lactose synthesis by altering gene expression [14]. Concomitant changes also occur in adipose and liver to support energetic and substrate demands of milk synthesis by the mammary gland. Lactation is metabolically demanding because nearly all the proteins and lactose in milk are synthesized in the mammary gland. Milk also provides over 50% of caloric energy to neonates as fat, and during lactation, the mammary gland surpasses the liver as one of the foremost lipid-synthesizing organs within the body [15–17]. Over the course of the typical three-week lactation period of mice, fatty acids produced by the mammary gland nearly equal the dam’s body weight of 32 g [17–19]. The fatty acids that make up triacylglycerol (TG) in milk are either absorbed from maternal circulation or synthesized *de novo*. To provide adequate milk fat to suckling neonates, mice fed a standard diet (8–10% kilocalories from fat) primarily rely on *de novo* synthesis of fatty acids from glucose and amino acids [17]. Maternal nutrition affects the dependence of the mammary gland on *de novo* synthesis or sourcing fatty acids from the blood, which reflects both dietary sources and mobilization from maternal adipose stores, with *de novo* lipogenesis lowered by high dietary fat content [20].

Understanding how maternal tissues respond to nutritional environment and modify dam metabolism may help in understanding how maternal health, nutrition, and behavior impact offspring growth and development. To this end, we conducted a series of studies with the ICR outbred line of mice and analyzed the impact of feeding a HF diet (60% kcal fat) on maternal physiology, behavior, and milk composition [21–25]. Relative to control (CON) diet, which was matched to the HF diet in sucrose, HF diet altered circadian patterns of maternal feeding behavior and elevated hair [23] and fecal corticosterone [25]. Dams on HF diet mobilized energy stores to support lactation, while CON mice primarily obtained energy for milk synthesis from increased food consumption [25]. Growth rate of litters was higher for HF diet dams with greater litter weight beginning on postnatal day four [24]. Higher litter growth rate of HF diet dams related to increased lactose content of milk, and although variable results were obtained, potentially also greater protein and lipid content of milk [21,23,25]. HF diet also changed the relative abundance of multiple fatty acids in milk of mice, decreasing the proportion of shorter chain and saturated groups and increasing fatty acyl chain length and unsaturation [22,25].

The mammary gland and liver are the two most metabolically active organs during lactation and require adequate turnover of proteins to support the high level of metabolism. Proteostatic mechanisms maintain cellular protein equilibrium through the continuous synthesis and degradation of proteins. Our previous study of the bovine mammary gland transcriptome during mid lactation found proteostatic associated mechanisms were central to supporting mammary function and milk synthesis [26]. The turnover rate of proteins varies with function, and can be affected by changes in nutrient supply and external environment of the organism [27–30]. Given the phenotypic responses of dams to HF diet, our objective was to determine maternal adaptation of global protein abundance and fractional synthetic rate (FSR) to HF diet in the liver and mammary gland of peak lactation mice. Label-free **s**hotgun proteomic analysis using liquid chromatography tandem mass spectrometry (LC-MS/MS) was used to measure protein abundance. To capture the impact of diet on FSR of proteins in liver and mammary tissue, animals were metabolically labeled with deuterium oxide (D_2_O) for approximately 24 hours prior to tissue collection and mass isotopomer distribution analysis (MIDA) of LC-MS/MS spectra was conducted [31]. Data were analyzed to 1) determine if protein abundance related to FSR; and 2) identify differentially abundant proteins (DAP) and proteins with different FSR in liver and mammary between dams fed a HF versus a CON diet.

## Materials and methods

### Animal care and treatments

**Fig 1:**
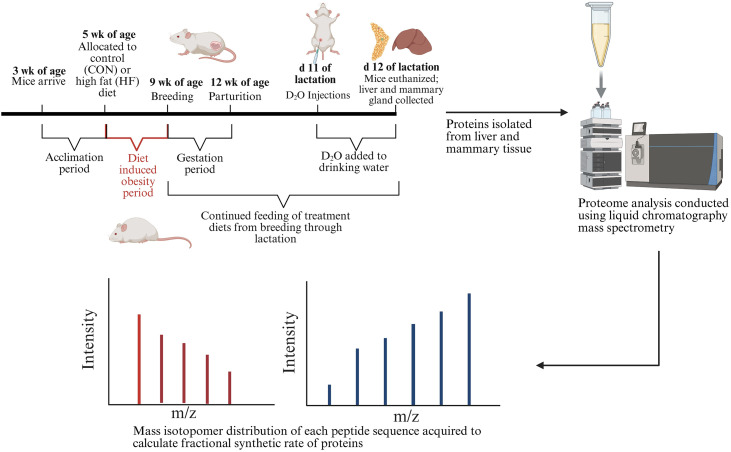
Experiment design and timeline for mice fed a high fat (HF) or control (CON) diet from week 5 to week 9 of age. Deuterium oxide (D_2_O) was administered 24 h before liver and mammary gland were collected on d 12 of lactation to measure the global proteome and fractional synthetic rate. Reprinted from Biorender.com under a CC BY license, with permission from Biorender.com, original copyright 2026.

The study protocol was reviewed and approved by Purdue University Animal Care and Use Committee (Protocol No. 2104002135) before beginning experiments. Briefly, three-week-old female ICR mice (n = 87) arrived at Purdue University from Envigo (Indianapolis, IN) in two separate cohorts (cohort 1 n = 42; cohort 2 n = 43). After two weeks of acclimation, mice were randomly assigned to experimental diets: CON (n = 36) or HF (n = 49). The tissue analyzed in this study was from five randomly selected mice on CON diets and five on HF diets. The subset of mice (n = 5 CON and n = 5 HF diet) that are the subject of the described study were exposed to 12 h of light and 12 h of dark, light-dark cycles, throughout the entire study. These mice were housed at Purdue University Centrally Managed Laboratory Animal Facilities in conventional cages with 3–5 animals per cage based on dietary treatments and fed *ad libitum* until 9 weeks of age. The 4-week period spanning from 5 weeks of age to 9 weeks of age, represents the experimental period for diet induced obesity of HF feeding, although the treatment diets were continued through gestation and lactation (Fig 1).

At nine weeks of age, female mice were bred at a 2:1 ratio with males and gave birth naturally. Males bred both HF and CON animals, therefore, our analyses assume that the male to which a dam was mated had no effect on the results. Within 36 h of birth, litter sizes were standardized to 8 pups/litter by cross-fostering within diet-light treatment or by euthanasia [24]. All animals were euthanized on lactation d 12 by slow fill CO_2_ inhalation, followed by cervical dislocation as a secondary method. The method of euthanasia is an American Veterinary Medical Association approved method [32].

The CON diet had 10% energy from fat, 20% energy from protein, and 70% energy from carbohydrates (Research Diet #D12450J; 3.85 kcal/g). The HF diet had 60% energy from fat, 20% from protein, and 20% from carbohydrates (Research Diet #D12492; 5.24 kcal/g). Diets had the same sucrose content. The ingredient composition of the diet can be found in previous publications [22,23] or on Research Diets, Inc. website.

### Deuterium oxide administration and sample collection

Optimal metabolic labeling for proteome MIDA studies is achieved at ~2.5% steady-state enrichment of D_2_O [31]. To reach this enrichment, doses were adapted from work of others [27], and n = 5 animals on each diet were administered 2 boluses of 20 µL/g of body weight IP of D_2_O (Millipore Sigma, Burlington, MA, USA) 4 h apart at 0800 and 1200 on d 11 of lactation with the first dose ~24 h prior to euthanasia. After the first injection, drinking water of mice was supplemented with 4% D_2_O (v/v) to maintain enrichment.

On d 12 of lactation, dams were separated from pups for 3 h and then anesthetized using 3% isoflurane gas at a rate of 1.0 L/minute of oxygen for milking. Following milking, dams were euthanized as described previously. Mammary gland number 4 was snap frozen in liquid nitrogen and stored at −80 °C until further analysis. Then, the entire liver was removed and the left lateral lobe was cut, portioned, snap frozen in liquid nitrogen, and stored at −80°C until further analysis.

### Analysis of deuterium oxide enrichment in mammary tissue samples

Due to limited plasma sample size remaining from previous analyses, D_2_O enrichment analysis was modified from [33] to measure deuterium labeled acetone extracted from mammary tissue. Analysis was performed in the Metabolite Profile Facility in Bindley Bioscience Center at Purdue University. Approximately 120 mg of tissue was weighed (40–184 mg) and placed into a 2 mL Precellys tissue homogenizer tube with CK28 ceramic beads inside the tube. Samples were homogenized using the Precellys 24 homogenizer (Bertin Corp. Rockville, MD) for one cycle at 6,500 rpm for 30 sec. Then, 20 µL of 10 N NaOH and 100 µL of 95:5 acetonitrile:acetone was added, and samples were mixed by vortexing for 10 min. Samples were incubated at room temperature (RT) for 24 h. Labeled acetone was extracted by adding 500 µL of chloroform and vortexing for 5 min, followed by centrifugation to collect the organic fraction, which was used for gas chromatography-mass spectrometry (GC-MS) analysis of D_2_O.

As previously described [34], a standard curve was generated by conducting a serial dilution of D_2_O with distilled water. The serial dilution generated a standard curve ranging from 16% D_2_O to 0% D_2_O. Similar to treatment of samples above, standards were combined with 2 µL of 10 N NaOH and 4 µL of 95:5 acetonitrile:acetone, centrifuged, and reacted overnight at RT. Then, 0.5 mL of 100% chloroform was added and vortexed, sample centrifuged at 14,000 rpm for 1 min at RT, then 300 µL of the supernatant was transferred to a GC-MS vial for analysis.

An Agilent 5975 C series GC/MSD system with an Agilent 7890 A (GC) and 7683 B injector was used to analyze each sample for unlabeled and labeled acetone (Agilent technologies, Santa Clara, CA). An Agilent Select FAME GC column (50 m x 0.25 mm, film thickness 0.25 um) was used (Agilent Technologies, Santa Clara, CA). The GC carrier gas was helium with a linear flow rate of 1.0 ml/min. The programmed GC temperature gradient was as follows: time 0–5 minutes, 60°C then ramped to 100°C at a rate of 20°C/minute, then ramped to 220°C at a rate of 50°C/minute with a 1-minute hold at the end. The total run time was approximately 10.5 minutes. The GC inlet was set to 250°C and samples were injected in split mode using a split ratio of 20:1. The MS source was set to 230°C. MS data were collected in select ion mode to look for acetone ions 58 and 59 m/z. All data were analyzed with Agilent Chemstation software (Version E.02). The acetone eluted from the column at 5.1 minutes.

### Measurement of β-hydroxybutyrate in liver tissue

Liver tissue from a subset of six mice per treatment from the larger study was used for analysis of BHB, a ketone body, to determine if HF diet induced ketogenesis. Analysis was performed in the Metabolite Profile Facility in Bindley Bioscience Center at Purdue University. Approximately 150 mg of liver tissue was homogenized for four 15 s intervals at 5,500 rpm in deionized water on a Precellys Evolution homogenizer using a Precellys CK14 tube. The homogenized sample was centrifuged for 10 min at 13,000 rpm at room temperature (RT). Then, 100 μL of supernatant was transferred to a new tube and combined with 10 μL of a 2 mg/10 μL in water solution of N-(3-Dimethylaminopropyl)-N′-ethylcarbodiimide hydrochloride (EDC) and 10 μL of a 6 M ^12^C-aniline solution that was diluted 50% with HCl. Samples reacted for 2 hours with gentle shaking at 400 rpm at RT, then the reaction was quenched using 3 uL of triethylamine. Samples were quickly vortexed after quenching, then centrifuged at 13,000 rpm for 5 min at RT. A stock solution of BHB standard (10 mg/mL) was diluted in deionized water to obtain 3.3ng/μL working standard. Then, 100 μL of working standard was labeled with 6 M ^13^C_6_-aniline in the same fashion as samples. Equal volume (20 μL) of ^12^C-aniline labeled sample and ^13^C_6_-aniline labeled standard were mixed and transferred into high performance liquid chromatography (HPLC) vials for analysis.

Samples were analyzed for BHB using liquid chromatography-mass spectrometry (LC-MS). An Agilent 1200 Rapid Resolution LC system coupled to an Agilent 6460 series QQQ MS was used to analyze BHB in each sample. A Waters Atlantis T3 (2.1 mm x 75 mm, 3um) column (Waters Corporation Milford, MA) was used for LC separation. HPLC grade water with 0.1% formic acid (v/v) was used as mobile phase A. HPLC grade acetonitrile with 0.1% formic acid (v/v) was used as mobile phase B. The linear LC gradient was as follows: time 0 min, 10% B; time 0.5 min, 10% B; time 12 min, 40% B; time 13 min, 98% B; time 14 min, 10% B; time 20 min, 10% B. The flow rate was 0.4 mL/min. Multiple reaction monitoring was used for MS analysis. The data were acquired in positive electrospray ionization (ESI) mode. The jet stream ESI interface had a gas temperature of 325°C, gas flow rate of 10 L/min, nebulizer pressure of 40 psi, sheath gas temperature of 250°C, sheath gas flow rate of 7 L/min, capillary voltage of 4000 V in positive mode, and nozzle voltage of 1500 V. The delta electron multiplier voltage was 400 V. The concentration of BHB (ng/mg) was determined from the relative response between ^12^C-aniline labeled sample verses ^13^C_6_-aniline labeled standard [35] and adjusted for sample dilution factors. All data were analyzed with Agilent Masshunter Quantitative Analysis (Version 10.1).

### Sample preparation and analysis of the global proteome

Protein sample preparation and LC-MS/MS analysis were performed in Purdue University’s Proteomics Profiling Facility in the Bindley Bioscience Center. Liver and mammary tissue were thawed and homogenized in 100 mM ammonium bicarbonate buffer at 6,500 rpm for 90 s in a bead beater (Bertin Technologies SAS, Paris, France), sonicated on ice, and isolated by centrifugation at 16,000 rpm for 10 min at 4°C. The bicinchoninic acid assay (Thermo Fisher Scientific, Waltham, MA, USA) was used to measure concentration of the isolated supernatant, and 50 µg of protein per sample was transferred to a microcentrifuge tube and −20°C acetone was added (1:4 v/v) to precipitate proteins overnight. Protein precipitate was collected by centrifugation at 13,500 rpm for 15 min at 4°C, dissolved in 8 M urea containing 10 mM dithiothreitol, and incubated at 37°C for 1 h to reduce disulfide bonds. To alkylate cysteines, samples were incubated for 1 h in the dark at RT with 97.5% acetonitrile, 0.5% triethyl phosphine, and 2% of iodoethanol, and then dried with a vacuum centrifuge. Dried sample was resuspended in 400 µL of 50 mM ammonium bicarbonate and digested with 80 µL of trypsin/LysC (Promega, Madison, WI, USA) under high pressure using a Barocycler (50^º^C, 60 cycles, 20,000 psi, Pressure Biosciences, Easton, MA, USA). Samples were desalted using MicroSpin columns (C18 silica, The Nest Group Inc., Ipswich, MA, USA), vacuum dried, and stored −80^º^C until analysis.

For LC-MS/MS analysis, samples were resuspended in 3% acetonitrile/0.1% formic acid at a final concentration of 1 µg/µL, and the Dionex UltiMate 3000 RSLC nano System (ThermoFisher Scientific, Odense, Denmark) connected to an Orbitrap Fusion Lumos Mass Spectrometer (Thermo Fisher Scientific, Waltham, MA, USA) was used. The A solvent contained 0.1% formic acid in water, and B consisted of 80% acetonitrile, 19.90% water, and 0.1% formic acid. Reverse-phase peptide separation was performed using a trap column (300 µm ID x 5 mm) packed with 5 µm 100 Å PepMap C18 medium coupled to a 50 cm long x 75 µm inner diameter analytical column packed with 2 µm 100 Å PepMap C18 silica (Thermo Fisher Scientific) at 50°C. One µL of sample was loaded into the column and collected from the analytical column at a flow rate of 200 nL/min with the aid of a 160 min LC gradient. The linear gradient from 2 to 27% of solvent B was 110 min, 27–40% of B for the next 15 min, 40–100% of B for the next 10 min, at which point the gradient was held at 100% of B for 10 min, before reverting to 2% of B, and held at 2% of B for 10 min for column equilibration. Furthermore, the column was washed and equilibrated using three 30 min LC gradient periods before loading the next sample. All data were acquired in the Orbitrap mass analyzer with the aid of a high-energy collision dissociation fragmentation scheme. For MS scans, the scan range was from 350 to 1600 m/z at a resolution of 120,000 while the automatic gain control target was set at 4 x 10^5^, maximum injection time 50 ms, dynamic exclusion 30 s, and intensity threshold 5.0 x 10^4^. MS data were acquired in data-dependent mode with a cycle time of 5 s/scan. Likewise, MS/MS data were acquired at a resolution of 15,000.

Raw data were imported into MaxQuant software version 1.6.3.3 for peptide matching to LC-MS/MS spectra, and proteins identified using the mouse Uniprot database. The following parameters were used for peptide identification: precursor mass tolerance of 10 ppm, enzyme specificity of trypsin/Lys-C enzyme allowing up to 2 missed cleavages, oxidation of methionine (M) as a variable modification, and iodoethanol (C) as a fixed modification. Mass Dynamics (massdynamics.com) was used for evaluation of MaxQuant aligned data by uploading the MaxQuant ProteinGroups.txt and summary.txt files. MaxQuant protein groups files (Table S2 in S1 Text liver and Table S3 in S1 Text mammary) were made public by uploading to the Purdue University Research Repository and can be retrieved using DOI: doi:10.4231/XX3A-7918 [36].

### Analysis of differentially abundant proteins

The relative label free quantification (LFQ) values were used for differential protein abundance analysis. Proteins selected for downstream analysis were found present (LFQ > 0) in 3 of 5 animals in at least one of the dietary treatment groups (Tables S4 and S5 in S1 Text). One-half the lowest LFQ values was used to replace data with a zero value, and then LFQ values were Log2 transformed prior to upload to Metaboanalyst 6.0 for statistical analysis and data visualization. Two sample t-tests were conducted in Metaboanalyst 6.0. Scores-plots of PCA and Ward method for hierarchical cluster analysis demonstrated by a heatmap with dendrogram were also generated using Metaboanalyst 6.0.

### Data processing for protein fractional synthetic rate

Data processing for protein FSR is outlined in Figure S1 in S1 Text. The raw LC-MS/MS spectral data were processed in MaxQuant software version 1.6.3.3 [37] to identify peptide sequences (PSM) and peptide-protein associations, and to extract data on observed isotopomer distributions (OID). They were then read into R for additional processing. After joining OID to PSM and protein associations, the data were filtered to exclude probable contaminants or reverse reads, and to retain only those cases for which at least one OID was available. If > 1 OID was available for a given PSM for a given sample, the OID in different scans were required to have Spearman rank correlations ≥ 0.8 to ensure inter-scan consistency, and only the scan resulting in the lowest posterior error probability score was retained for analysis. As an additional quality control measure, we also excluded any PSM for which < 50% of the samples had OID. All proteins or protein groups with at least 1 uniquely associated PSM were analyzed.

We estimated FSR (k, in d^-1^) for each protein by minimizing the sum-of-squared error between the observed and expected isotopomer distributions (EID) for peptides associated with the protein. EID were calculated as m_t_ = (1 – f_t_)m_0_ + f_t_m_∞_, where f_t_ is the proportion of labeled peptide at observed labeling time t, m_t_ is the EID time t (in days), m_0_ is the theoretical EID prior to labeling, and m_∞_ is the asymptotic, labeled EID. Theoretical baseline and asymptotic EID were calculated based on Table 1 in [31], taking amino acid sequences and body water enrichment (Table S1 in S1 Text) as given. Non-enriched isotopic abundances were based on [38]. We assumed a single-compartment exponential model for peptide turnover, so that the fraction of labeled peptide was calculated as f_t_ = 1 − e^(−kt)^. The rate k was estimated on the log scale using the Levenberg–Marquardt algorithm (package nls.multstart) [39]. Asymptotic confidence intervals were calculated from Fisher information by the delta method after multiplying the log-scale standard error by the number of peaks in the OID to adjust for the non-independence of errors within a given PSM (Figure S2 in S1 Text).

Data were filtered by confidence intervals to maintain FSR values within the dataset that were estimated. Estimates were excluded if the algorithm did not converge, the confidence interval associated with the protein group was missing, or if the confidence interval spanned from negative to positive values suggesting poor reliability of the FSR calculation (Table S6 in S1 Text liver and Table S7 in S1 Text mammary gland). Data were then filtered by similar lead protein. If protein groups had similar lead protein, then the group with the smallest number of proteins was used to avoid contamination of the FSR estimate by other proteins. The duration of label administration (t) for this experiment was 1 d. The lower bound at which f can be reliably estimated is determined by the analytical threshold of the instrument, and the upper bound is determined by the advancement of labeled newly synthesized proteins towards plateau. Using the guidelines in [31], the range of f used in our experiment was 5% to 80%. The range of FSR (k) that aligned with an f value of 5% and 80% was 0.06 d^-1^ and 2 d^-1^ [31], respectively, describing the lower and upper bounds of expected FSR values, which were used to categorically evaluate FSR estimates into fastest (> 2 d^-1^), slowest (< 0.06 d^-1^), and intermediate (0.6 d^-1^ to 2 d^-1^).

### Data analysis to determine the relationship between protein abundance and FSR in liver and mammary gland of CON mice

To investigate potential relationships between protein abundance and FSR in the liver and mammary gland of CON mice, each tissue’s dataset was divided into thirds corresponding to highest/fastest, intermediate, and lowest/slowest abundant proteins or FSR values. This resulted in the following categories for abundance: highest third (0.33), intermediate (0.66), and lowest third (0.99). Similarly, FSR data were divided into the fastest third (0.33), intermediate third (0.66), and slowest third (0.99). Venny 2.1.0 [40] was used to query for proteins overlapping in abundance and FSR datasets.

### Differential analysis of FSR values

For differential analysis of FSR values between HF and CON (Tables S8 and S9 in S1 Text), data were removed if the difference in the FSR estimate was outside of the measurable range of 0.06 to 2, if the confidence interval for the differential analysis associated with the protein group was lacking, or if the confidence interval of the differential analysis included 0, then there was no significant difference. FSR estimates were compared between HF and CON using a two-sample t-test with correction for multiple testing. A raw *P*-value cut-off < 0.05 was used to identify proteins with differential FSR. In instances with duplicate lead proteins with multiple FSR estimates, FSR data with the least number of proteins was kept for downstream analysis. If the protein group had the same number of proteins, then the protein group with the smallest confidence interval range was kept.

### Functional annotation analysis

After data were analyzed to determine DAP and differential FSR increased and decreased due to HF diet, functional annotation cluster analysis was conducted in Database for Annotation, Visualization and Integrated Discovery (DAVID) [41]. Uniprot ID of the major lead protein of both protein abundance and FSR data was converted into official gene symbol using either DAVID or Synaptic Gene Ontologies (SynGO) [42]. Official gene symbol data was used for functional annotation cluster analysis in DAVID [41].

### Measurement of total and phosphorylated Mammalian Target of Rapamycin (mTOR)

Liver tissue from a subset of five mice per treatment were used to investigate whether total mTOR or phosphorylated (phospho) mTOR were altered in response to HF diet. The manufacturer’s protocol of the phospho-mTOR and total mTOR ELISA kit (ab279869 Abcam, Cambridge, UK) was followed with the following adaptations. Liver tissue was homogenized in 1X cell lysis buffer provided in the mTOR ELISA kit for 30 s using a Fisherbrand Homogenizer 150 (FisherScientific, Waltham, MA). Protein concentration per well was standardized to 150 ug. Samples were analyzed at 450 nm using a Spark 10M spectrophotometer and data are presented as absorbance at 450 nm· 150 ug of protein^-1^.

### Statistical analysis of liver BHB and mTOR

Data from analysis of liver BHB and mTOR were analyzed using a linear model (PROC GLM) in SAS 9.4, and least squares means were generated using the “means” function in SAS.

## Results

### Impact of high fat diet on neonate growth and milk composition

The findings presented in this manuscript represent a component of a larger study [23–25] for which mice were fed a HF or CON diet from 5 weeks to 9 weeks of age to achieve divergent weight and percent body fat prior to mating (Fig 1). The CON diet had 10% energy from fat, 20% from protein, and 70% energy from carbohydrates (3.85 kcal/g). The HF diet had 60% energy from fat, 20% from protein, and 20% from carbohydrates (5.24 kcal/g). After 4 weeks of feeding, mice on HF diet weighed more (P < 0.001) and had higher percent body fat (P = 0.05) than those on CON diets [23–24]. Although mice fed the HF diet ingested more kcal, the total amount of intake (g of food) was lower than CON (P **<** 0.01) and timing of intake was altered, with HF consuming more food during the day (P < 0.05) than mice on CON diet, which is contrary to their normal nocturnal behavior [23–24]. Fecal circadian sampling over 48 h demonstrated that HF mice had elevated basal corticosterone and attenuated circadian rhythms [23–24]. Taken together, these responses support that HF diet disrupted normal behavior and physiology.

Although birth weights were similar between pups born to HF and CON mice, by postnatal day 4, pups in litters (standardized to 8 pups/dam by cross-fostering within treatment or by euthanasia) of HF dams weighed significantly more than those in CON litters [24]. The difference in weight between pups of HF and CON dams remained until the termination of the study on lactation day 12 when milk, mammary glands, and liver were collected for analysis. Although in this cohort of mice, lactose and protein content of lactation d 12 milk was similar between CON and HF dams [24], our previous experiments with similar study designs found HF diet increased lactose, fat, and protein content of milk [21,22,25]. Analysis of lipid profiles of serum, mammary tissue, and milk for the current and previous studies found HF significantly increased the length and number of unsaturated bonds of fatty acyl chains in triglyceride molecules and decreased the proportion of shorter chain and saturated groups [22,25].

### Analysis of global proteome data quality and selection of proteins for downstream analysis

To understand how maternal metabolic adaptations to HF diet may be driving differences in litter growth and milk composition, global protein abundance and FSR were measured in the liver and mammary gland of mice collected on lactation d 12. Global protein abundance was captured using label-free shotgun proteomic analysis with LC-MS/MS. To metabolically label newly synthesized proteins, mice were dosed with D_2_O 24 h prior to euthanasia and tissue collection. Analysis of body water enrichment with D_2_O found a mean of 3.1% D_2_O enrichment across all treated mice (Supplemental Table S1 in S1 Text; all supplemental data are available through the Purdue University Research Repository DOI: doi:10.4231/XX3A-7918 [36]), which is an optimal level for measuring FSR of the global proteome using MIDA of LC-MS/MS spectra [31].

Prior to imputation and analysis, quality of proteome data (liver, Supplemental Fig S3 in S1 Text and mammary, Supplemental Fig S4 in S1 Text) was assessed using correlation analysis of LFQ intensity values between samples. Within each tissue type, LFQ values were highly correlated across all samples with mean *r* = 0.96 for liver and *r* = 0.94 for mammary. Moreover, the overall proportion of missing LFQ values and the proportion of proteins with missing LFQ values were similar across samples within each tissue and treatment group. Together, supporting good quality data for further analysis.

### Number of proteins used for downstream analysis in abundance and FSR datasets for liver and mammary gland

For analysis of abundance, proteins with LFQ intensity values detected in at least 3 of 5 samples in one of the dietary treatments were selected for downstream analysis resulting in 1,830 proteins considered for analysis for liver and 1,261 for mammary gland. Proteins with FSR values between > 0.06 and < 2 were considered to have sufficient quality estimates of FSR [31] for downstream analysis of differential FSR between diet groups (c.f. Figure S2 in S1 Text). Proteins with FSR point estimates >2 were categorized as the fastest and those with FSR point estimates < 0.06 were categorized as proteins with slowest synthetic rate. In these cases, a numerical FSR value cannot be estimated with precision (see Figure S2B in S1 Text). However, the lack of precision occurs because the observed protein mass isotopomer distribution converges on either the unlabeled condition, in which no protein molecules are newly synthesized (i.e., slow FSR), or on the asymptotic condition in which all proteins have been replaced during the labeling period (i.e., fast FSR).

In liver of CON and HF diet groups, 25 and 6 proteins, respectively, had FSR > 2,190 and 63 had FSR < 0.06, and 2069 and 1886 non-duplicate proteins had FSR ranging between > 0.06 to < 2. In mammary of CON and HF groups, 3 and 1 proteins, respectively, had FSR > 2.0, 71 and 54 with FSR < 0.06, and 1225 and 1111 non-duplicate proteins with FSR > 0.06 to < 2.

### Analysis of the relationship between protein abundance and FSR in liver and mammary glands of CON diet fed mice

Two approaches were used to determine if protein abundance related to FSR in the liver and mammary gland of CON mice, the first being correlation analysis. In the liver of CON mice, correlation analysis of the 1081 proteins with both log base 2 LFQ values and FSR ranging > 0.06 to < 2 found *r* = −0.33, *P* < 0.0001, and R^2^ = 0.11. Similarly, correlation analysis of the 705 mammary gland proteins in CON mice with both FSR and abundance data found *r* = −0.23, *P* < 0.0001, and R^2^ = 0.05. Demonstrating there was an overall negative relationship between protein abundance and FSR and that 11% and 5% of the variation in the data can be accounted for by the negative relationship between abundance and FSR.

**Fig 2:**
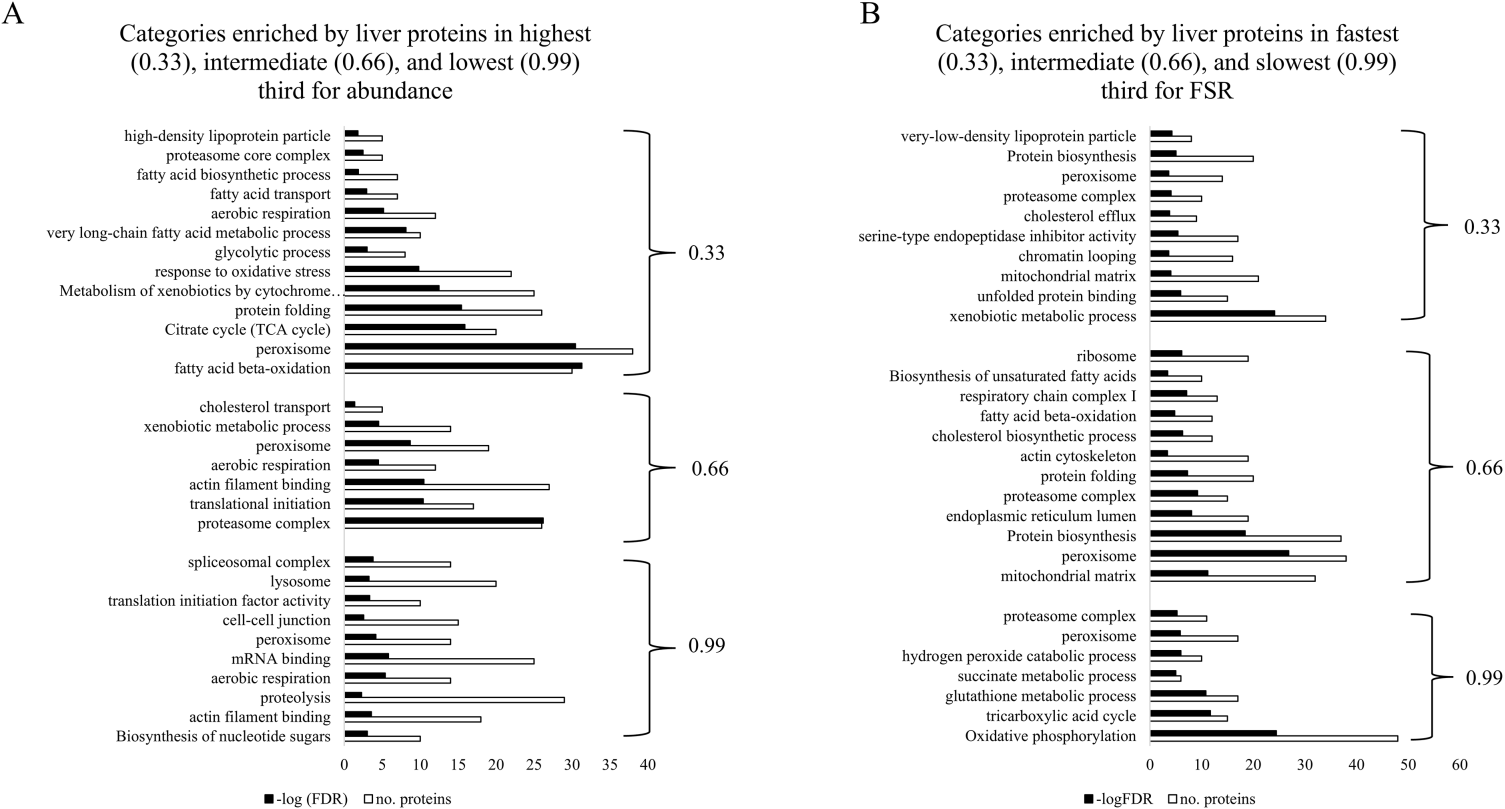
Functional annotation categories enriched by liver proteins in highest/fastest third (0.33), intermediate third (0.66), and lowest/slowest third (0.99) for A) abundance or B) fractional synthetic rate (FSR). Abundance and FSR data were categorized into three groups based on highest abundance and fastest FSR (0.33), intermediate abundance and FSR (0.66), and lowest abundance and slowest FSR (0.99), then functional annotation analysis was conducted of those groups to determine molecular signatures within the categories.

**Fig 3:**
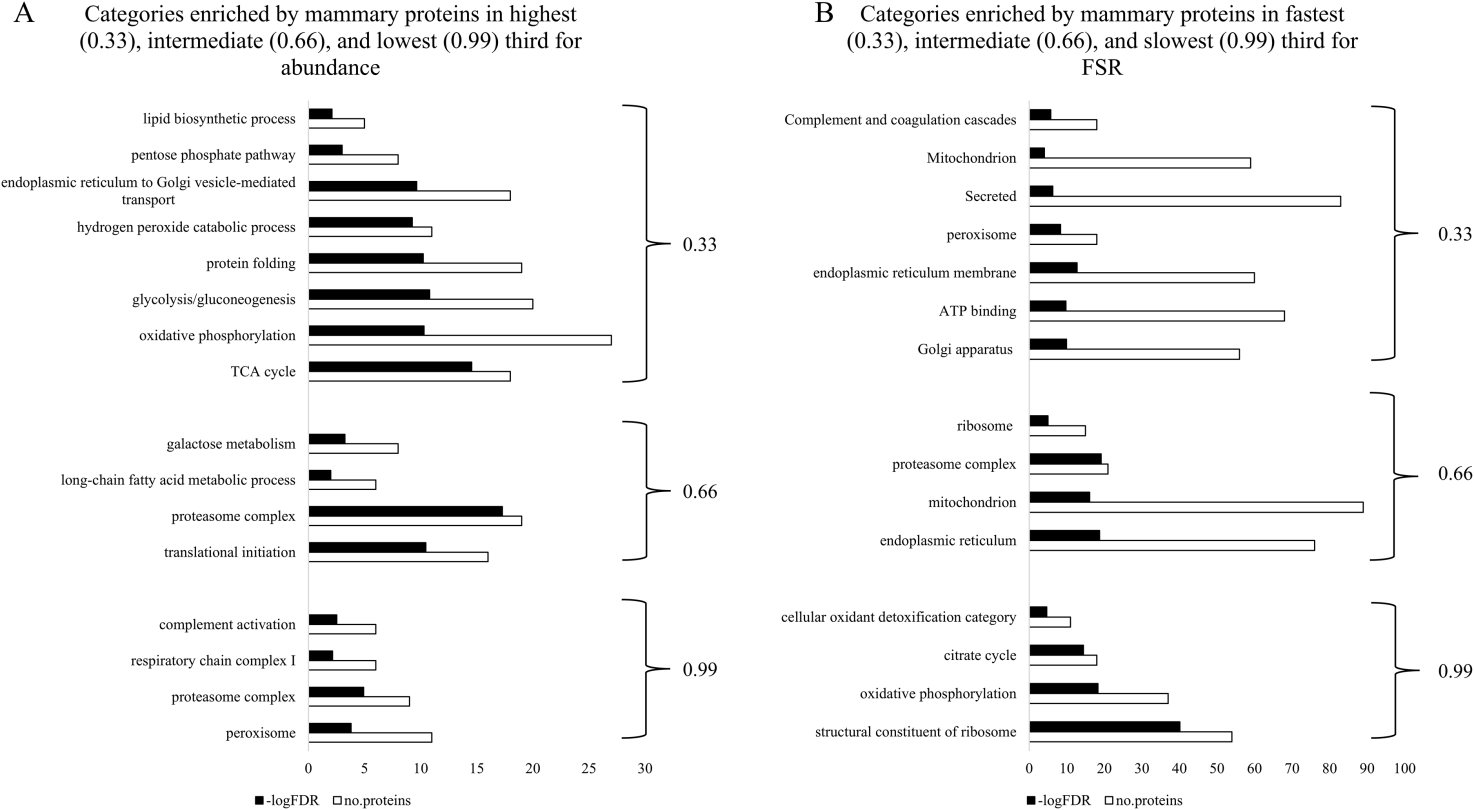
Functional annotation categories enriched by mammary gland proteins in highest/fastest third (0.33), intermediate third (0.66), and lowest/slowest third (0.99) for A) abundance or B) fractional synthetic rate (FSR). Abundance and FSR data were categorized into three groups based on highest abundance and fastest FSR, intermediate abundance and FSR, and lowest abundance and slowest FSR, then functional annotation analysis was conducted of those groups to determine molecular signatures within the categories.

To further investigate relationships between protein abundance and FSR, LFQ and FSR data were divided into thirds to represent high/fast (0.33), intermediate (0.66), and low/slow (0.99) categories of abundance or FSR values, respectively (Table 1; Figs 2 and 3). Analysis of overlap between 0.33 categories of liver proteins by abundance and FSR for CON animals found of the proteins common between the FSR and abundance data, only 90 (6%) proteins were in the highest third for abundance and fastest third for FSR (0.33 and 0.33; Table 1). Functional annotation analysis of the 90 proteins overlapping in the highest third for abundance and fastest third for FSR categories (0.33 and 0.33) in liver of CON mice found they enriched xenobiotic metabolic process, high-density lipoprotein particle, and unfolded protein binding (Table 2). Liver proteins that were in the highest third for abundance and slowest third for FSR (0.33 abundance and 0.99 FSR) enriched cytosolic ribosome, citrate cycle, response to oxidative stress, and fatty acid metabolism. Liver proteins that were in the lowest third for abundance and fastest third for FSR categories (0.99 abundance and 0.33 FSR) enriched oxidoreductase activity, proteolysis and lipid biosynthesis. Liver proteins in the lowest third for abundance and slowest third for FSR (0.99 and 0.99) enriched transit peptide and endoplasmic reticulum categories.

**Table 1:** Number of proteins overlapping between abundance and fractional synthetic rate (FSR) in liver and mammary gland of mice fed the control (CON) diet. Abundance and FSR data of CON mice were categorized into three groups based on highest abundance and fastest FSR (0.33), intermediate abundance and FSR (0.66), and lowest abundance and slowest FSR (0.99). Proteins were evaluated for overlap between highest third (0.33), intermediate third (0.66) and lowest third (0.99) in abundance and fastest third FSR (0.33), intermediate third (0.66), and slowest third FSR (0.99).

Liver				
FSR				
Abundance	0.33	0.66	0.99	no overlap
0.33	90	179	252	88
0.66	163	193	155	99
0.99	203	162	93	152
no overlap	237	159	194	
Mammary gland				
FSR				
Abundance	0.33	0.66	0.99	no overlap
0.33	86	114	154	66
0.66	106	152	108	54
0.99	121	116	62	121
no overlap	192	123	181	

**Table 2:** Categories enriched by liver proteins overlapping between highest/fastest third for abundance and fractional synthetic rate (FSR) (0.33 and 0.33), highest for abundance (0.33) and slowest third for FSR (0.99), lowest for abundance (0.99) and fastest for FSR (0.33), and lowest for abundance (0.99) and slowest FSR (0.99). Abundance and FSR data were categorized into three groups based on highest abundance and fastest FSR (0.33), intermediate abundance and FSR (0.66), and lowest abundance and slowest FSR (0.99), then functional annotation analysis was conducted to evaluate similar molecular signatures between groups of interest.

Term	Genes	FDR
**0.33 abundance and 0.33 FSR**		
GO:0006805 ~ xenobiotic metabolic process	CYP2C69, TPMT, CYP2D10, CYP2A12, GSTP1, CYP3A44, CYP2C40, FMO1, CYP2F2, CYP2C29, UGT1A6B, CYP2D26	1.2E-11
GO:1990904 ~ ribonucleoprotein complex	RPL4, DDX5, RPS9, HNRNPK, RPS27, HNRNPF, ACTN4, FAU, RPS11, RPS27A	4.0E-06
GO:0005789 ~ endoplasmic reticulum membrane	CYB5A, HSPA5, RDH9, SAR1B, SURF4, EPHX1, CYP3A44, CYP3A11, FMO1, CYP2C29, UGT1A6B, CYP2D26, PGRMC1, CYP2D10, CYP2A12, CYP2C40, CYP2F2, HPD, SLC27A2	1.4E-07
GO:0034364 ~ high-density lipoprotein particle	APOH, PON1, HDLBP, APOA1, APOA4	3.8E-05
GO:0051082 ~ unfolded protein binding	DNAJA1, CCT2, HSP90AB1, HSPA5, CCT8	3.2E-03
mmu00980:Metabolism of xenobiotics by cytochrome P450	GSTM1, GSTP1, EPHX1, CYP2F2, UGT1A6B	3.8E-02
KW-0809 ~ Transit peptide	NADK2, ALDH2, HIBADH, GPAM, ECI1, NDUFS3, ACO2, NIPSNAP1	4.1E-02
GO:0042730 ~ fibrinolysis	FGA, FGG, PLG	4.2E-02
**0.33 abundance and 0.99 FSR**		
GO:0005739 ~ mitochondrion	DMGDH, PECR, MTCH2, NDUFA13, ACAA2, ISOC2A, CISD1, RPS14, MPC2, HADH, GSTK1, SDHC, HOGA1, SDHA, SDHB, ALDH3A2, ACLY, ETHE1, GPD2, GPD1, UQCRC1, VDAC3, SUCLG2, VDAC1, SUCLG1, ALDH7A1, GCDH, MAOB, SHMT2, MTARC1, MGST1, AK2, GLYAT, ACAT3, ACAT2, ACAT1, PRDX3, PRDX2, ADH1, PRDX4, CYB5R3, GRPEL1, PRDX1, DECR1, HSPA9, MPST, GOT2, PRDX6, ACSF2, CS, GLUD1, ALDH6A1, AMACR, CPS1, UQCRQ, CYCS, ECHDC2, UBA1, TPP1, SLC25A1, SLC25A3, ABCD3, COX4I1, DBI, ETFA, ETFB, AIFM1, ACADL, NNT, KYNU, ME1, DLAT, ACADS, HIBCH, AASS, BCKDHB, SORD, HADHB, HADHA, SLC25A15, ALDH5A1, TST, PCCA, BDH1, IVD, EHHADH, SLC25A10, HAGH, AGXT, SLC25A5, DLD, SLC25A13, ALDH9A1, ECHS1, PCX, ETFDH, DLST, HSD17B10, BPHL, HSPD1, HMGCL, RPS15A, ALDH1B1, RPS3, SLC25A20, NDUFA9, CYB5B, BCAP31, NDUFA8, MDH1, MDH2, COX6C, HSPE1, SOD2, ASS1, SOD1, AGMAT, ALDH4A1, SUCLA2, GSTA4, SARDH, OTC, UOX	2.8E-78
GO:0022626 ~ cytosolic ribosome	RPL5, RPL3, RPL10, RPL32, RPL12, RPLP0, RPL8, RPL9, RPL6, RPL7, RPS4X, RPS14, RPL7A, RPS17, RPS15A, RPS16, RPL18A, RPS19, RPL14, RPS3, RPL13, RPL35, RPL38, RPS2, RPL15, RPL18, RPS10, RPL39, RPS13, RPL19, RPS7, RPS8, RPL23, RPL22, RPSA, RPL23A, EEF1A1, RPS25, RPL24, RPS20, RPS21, RPS23	9.1E-54
mmu00280:Valine, leucine and isoleucine degradation	ECHS1, ACAA2, BCKDHB, HSD17B10, ACAT3, ACAT2, ACAT1, HADHB, ALDH3A2, HMGCL, HADHA, ALDH6A1, PCCA, ALDH1B1, EHHADH, IVD, HADH, DLD, ALDH7A1, ACADS, HIBCH, ALDH9A1	1.4E-19
mmu00640:Propanoate metabolism	HADHA, ALDH6A1, ECHS1, SUCLA2, PCCA, EHHADH, BCKDHB, SUCLG2, SUCLG1, ACADS, DLD, HIBCH	2.6E-10
mmu00020:Citrate cycle (TCA cycle)	PCX, MDH1, MDH2, DLST, SDHC, SDHA, SDHB, CS, ACLY, SUCLA2, SUCLG2, SUCLG1, DLAT, DLD	5.6E-13
GO:0022627 ~ cytosolic small ribosomal subunit	RPS7, RPS8, RPSA, RPS4X, RPS25, RPS14, RPS17, RPS15A, RPS16, RPS19, RPS3, RPS20, RPS2, RPS21, RPS10, RPS13, RPS23	1.7E-18
GO:0033539 ~ fatty acid beta-oxidation using acyl-CoA dehydrogenase	GCDH, ACADL, IVD, ETFDH, ETFA, ETFB, ACADS	3.0E-08
DOMAIN:Aldehyde dehydrogenase	ALDH4A1, ALDH3A2, ALDH6A1, ALDH5A1, ALDH1B1, ALDH1A1, ALDH7A1, ALDH1A7, ALDH9A1	2.2E-10
GO:0022904 ~ respiratory electron transport chain	AIFM1, ETFDH, ETFA, ETFB, SOD2, SDHA, SDHB	7.3E-06
GO:0006105 ~ succinate metabolic process	ALDH5A1, SUCLA2, SUCLG2, SUCLG1, SDHA, SDHB	8.0E-08
mmu00980:Metabolism of xenobiotics by cytochrome P450	GSTK1, GSTM3, UGT2B34, UGT1A1, UGT2B36, UGT2B1, GSTO1, MGST1, GSTT1, HSD11B1, ADH1, GSTA4, UGT1A5, UGT2B5	5.6E-08
GO:0006979 ~ response to oxidative stress	ALAD, PRDX3, PRDX2, PRDX4, AIFM1, PRDX1, NAPRT, ALDH1A1, ETFDH, SOD2, PRDX6, SOD1	4.3E-06
GO:0005777 ~ peroxisome	ALDH3A2, PECR, GSTK1, HMGCL, ABCD3, AMACR, EHHADH, TKT, AGXT, ACOT4, UOX, SOD1	2.1E-07
mmu00250:Alanine, aspartate and glutamate metabolism	ALDH4A1, GLUD1, ALDH5A1, CPS1, GOT1, GOT2, ASL, AGXT, ASS1	7.7E-06
KW-0276 ~ Fatty acid metabolism	PECR, ECHS1, ACAA2, HSD17B10, ACSF2, ACAT1, HADHB, ALDH3A2, HADHA, ACADL, EHHADH, ECHDC2, HADH, ACADS, ACOT4, DECR1	3.0E-09
GO:0003985 ~ acetyl-CoA C-acetyltransferase activity	HADHB, ACAA2, ACAT3, ACAT2, ACAT1	8.6E-06
mmu00062:Fatty acid elongation	HADHB, HADHA, ECHS1, ACAA2, HADH, ACOT4	1.1E-03
mmu01230:Biosynthesis of amino acids	TPI1, PCX, GOT1, SHMT2, PGAM1, GOT2, ASS1, CS, CPS1, CTH, PGK1, ASL, TKT, OTC	8.2E-08
GO:0000050 ~ urea cycle	CPS1, ASL, ASS1, OTC, AGMAT	6.8E-05
GO:0009055 ~ electron transfer activity	ETFDH, CYCS, SDHC, ETFA, ETFB, SDHA, SDHB	3.9E-06
GO:0042776 ~ proton motive force-driven mitochondrial ATP synthesis	NDUFA9, NDUFA8, NDUFA13, SDHC, SDHA, SDHB	5.4E-03
KW-0049 ~ Antioxidant	PRDX3, PRDX2, PRDX4, PRDX1, PRDX6, SOD1	2.9E-05
GO:0000302 ~ response to reactive oxygen species	ALDH3A2, PRDX1, SOD2, PRDX6, SOD1	1.9E-03
GO:0019430 ~ removal of superoxide radicals	PRDX2, PRDX1, SOD2, SOD1	8.8E-03
KW-0249 ~ Electron transport	CYB5B, NDUFA9, NDUFA8, NDUFA13, ETFDH, SDHC, ETFA, ETFB, SDHA, SDHB, UQCRQ, UQCRC1, CYCS	3.6E-08
GO:0006749 ~ glutathione metabolic process	GSTM3, GSTK1, GSTA4, GSTO1, ETHE1, GLO1, CTH, MGST1, GSTT1, HAGH, SOD2, SOD1	1.5E-10
GO:0004032 ~ aldose reductase (NADPH) activity	AKR1C14, AKR1C13, AKR1D1, AKR1A1	1.1E-02
GO:0042776 ~ proton motive force-driven mitochondrial ATP synthesis	NDUFA9, NDUFA8, NDUFA13, SDHC, SDHA, SDHB	5.4E-03
**0.99 abundance and 0.33 FSR**		
GO:0016491 ~ oxidoreductase activity	VAT1, CYP2C37, ADH4, CYP2D9, DHRS7, AMBP, CYP2B10, AOX1, CYP3A13, CYP3A16, FADS1	1.3E-04
GO:0006508 ~ proteolysis	CTSA, USP47, ENPEP, USP4, ABHD5, PEPD, CLPX, CASP6, CASP3, PSME3, RNPEP, LONP2, PMPCB, PMPCA, MBL2	2.1E-02
GO:0016705 ~ oxidoreductase activity, acting on paired donors, with incorporation or reduction of molecular oxygen	CYP2D9, CYP4A10, FADS1, CYP4F13	7.5E-02
GO:0005524 ~ ATP binding	TOP2B, MVK, UBA6, ABCB4, VPS4B, UBE2D3, GMPS, DDX21, YARS2, MAT2A, RUVBL2, KIF5B, MYO18A, LONP2, CSK, ABCF2, ATAD1, DDX17, MAP2K1, IDH3G, ENTPD5, ACSL4, CLPX, CAPRIN1, TOP1, SNRNP200	2.3E-03
GO:0005787 ~ signal peptidase complex	SPCS2, SPCS1, SEC11A	2.7E-02
GO:0016485 ~ protein processing	ENPEP, DDI2, CASP3, RNPEP, LONP2, PMPCB, PMPCA	2.1E-02
GO:0006695 ~ cholesterol biosynthetic process	MVK, CES1E, LIPA, LSS, FDFT1	2.9E-02
GO:0002020 ~ protease binding	SERPINA1B, SERPINB6A, CASP3, SERPINF2, MUG2, LONP2, MBL1, MBL2	5.3E-03
KW-0496 ~ Mitochondrion	STOML2, GFM1, POLDIP2, NDUFB6, RAP1GDS1, MRPL39, DDX21, MRPS30, CLU, YARS2, PMPCB, PMPCA, FADS1, VAT1, ATAD1, AMBP, HSD3B3, IDH3G, CMC1, SIRT5, ACSL4, CLPX, SIRT3, ETNPPL, TMX2	2.2E-02
GO:0051015 ~ actin filament binding	CYFIP1, LASP1, DBNL, FLII, ARPC1A, MYO18A, CTNNA2, CNN3	2.3E-02
**0.99 abundance and 0.99 FSR**		
KW-0496 ~ Mitochondrion	TOMM40, NDUFA11, MAOA, MRPS34, COX7A2, CLYBL, MRPL10, APOO, BCL2L13, CHCHD3, SAMM50, SLC25A23, PDK1, AADAT, MTX2, ADHFE1, NDUFA2, ACOT13, BCS1L, GFER, TMCO1, COQ3, LYRM4, NDUFS6, L2HGDH, SSBP1, RTN4IP1, FXN	6.2E-11
KW-0809 ~ Transit peptide	COQ3, AADAT, NDUFS6, ADHFE1, COX7A2, L2HGDH, SSBP1, RTN4IP1, FXN, CLYBL, MRPL10, PDK1	8.0E-05
TRANSIT:Mitochondrion	COQ3, AADAT, NDUFS6, ADHFE1, COX7A2, L2HGDH, SSBP1, RTN4IP1, FXN, CLYBL, MRPL10, PDK1	2.1E-03
GO:0005783 ~ endoplasmic reticulum	FKBP2, TMED9, SDF2L1, RTN4, TMCO1, CES2A, UFL1, ASPH, RER1, EMC2, MLEC, GNPNAT1, RDH14, DHCR7, CLIC1, TMED4	4.4E-04
KW-0256 ~ Endoplasmic reticulum	FKBP2, TMED9, SDF2L1, RTN4, TMCO1, CES2A, UFL1, APOO, ASPH, DAD1, EMC2, MLEC, DHCR7, CLIC1, TMED4	1.3E-02

Of the proteins common between the FSR and abundance data sets for mammary gland of CON animals, only 86 (8%) were categorized as highest third for abundance and fastest third for FSR (0.33 and 0.33; Table 1). Nine of the 86 mammary gland proteins overlapping for 0.33 abundance and 0.33 FSR were secreted in milk (e.g., CSN1S2B, WAP, CSN1S2A, CSN3, CSN1S1, CSN2,LTF, MFGE8, XDH) and enriched both the milk protein and extracellular space categories (Table 3; Fig 3). Proteins in the highest third for abundance (0.33) and slowest third for FSR (0.99) enriched the cytosolic ribosome category, functioned as enzymes in the citrate cycle, electron transport/respiratory chain, and in mediating fatty acid β-oxidation as well as catabolism of hydrogen peroxide (Table 3). Proteins in lowest third for abundance (0.99) and fastest third for FSR (0.33) enriched peroxisome, mRNA splicing, and complement activation categories (Table 3). Mammary proteins both in lowest/slowest third for abundance and FSR categories (0.99 and 0.99) enriched oxidative phosphorylation and ER-Golgi transport (Table 3). Together, linear regression analysis and analysis for overlap between highest/fastest, intermediate, and lowest/slowest categories of protein abundance and FSR demonstrate very few positive or small-moderate negative relationships between these variables in the liver and mammary gland of CON mice.

**Table 3:** Categories enriched by mammary gland proteins overlapping between highest/fastest third for abundance and fractional synthetic rate (FSR) (0.33 and 0.33), highest for abundance (0.33) and slowest third for FSR (0.99), lowest for abundance (0.99) and fastest for FSR (0.33), and lowest third for abundance (0.99) and slowest FSR (0.99). Abundance and FSR data were categorized into three groups based on highest and fastest FSR (0.33), intermediate abundance and FSR (0.66), and lowest abundance and slowest FSR (0.99), then functional annotation analysis was conducted to evaluate similar molecular signatures between groups of interest.

Term	Genes	FDR
**0.33 abundance and 0.33 FSR**		
KW-0494 ~ Milk protein	CSN1S2B, WAP, CSN1S2A, CSN3, CSN1S1, CSN2	5.40E-09
GO:0005615 ~ extracellular space	WAP, CSN3, CSN2, CLU, C3, SERPINA1B, HPX, TTR, APOH, QSOX1, CSN1S1, GC, XDH, CTSD, CTSB, FGB, AHSG, SERPINA3K, CSN1S2A, APOA1, APOA4, CEL, CP, CES1C, CSN1S2B, ALB, MFGE8, ALDOA, NUCB2, LTF	1.78E-11
mmu00620:Pyruvate metabolism	LDHA, PKM, ALDH2, MDH1, MDH2, ALDH7A1, ACACA	6.00E-05
GO:0051287 ~ NAD binding	LDHA, ALDH2, MDH1, GPD1, NDUFS2, NDUFV1	3.39E-05
GO:0034364 ~ high-density lipoprotein particle	APOH, HDLBP, APOA1, APOA4	0.0014
GO:0008289 ~ lipid binding	C3, APOH, ALB, APOA1, APOA4, CD36	0.01463
GO:0042470 ~ melanosome	HSPA8, HSP90AB1, ATP6V1B2, SLC3A2, CTSD, RAN, CTSB, HSP90B1	4.99E-07
KW-0931 ~ ER-Golgi transport	BCAP31, ARF4, RTN3, ARF1, SEC23B, ARCN1	6.77E-04
GO:1990904 ~ ribonucleoprotein complex	HNRNPL, RPS25, SYNCRIP, HSPA8, HNRNPK, HNRNPF, IQGAP1, RPS27A, RPL18, EEF2	3.21E-06
**0.33 abundance and 0.99 FSR**		
GO:0022626 ~ cytosolic ribosome	RPL4, RPL5, RPL3, RPL10, RPL32, RPL31, RPL12, RPL11, RPLP0, RPL8, RPL10A, RPL6, RPL7, RPS4X, RPS15, RPS14, RPL7A, RPS17, RPS15A, RPS16, RPL18A, RPS19, RPL14, RPL13, RPL35, RPS2, RPL15, RPS13, RPL19, RPS7, RPL23, RPS6, RPL22, RPSA, RPL23A, EEF1A1, RPL27A,	9.19E-70
GO:0005739 ~ mitochondrion	SLC25A1, MTCH2, SLC25A3, ACAA2, COX4I1, NDUFA10, ETFA, RPL10A, PHB2, GMPPB, RPS14, MPC1, MPC2, ME1, DLAT, ACADM, HADH, IDH3A, GPX1, SDHA, SDHB, TUFM, ACLY, BDH1, IVD, UQCRC1, VDAC1, UQCRC2, SLC25A5, DLD, BCAT2, ECHS1, PCX, MTARC2, GLRX, PDHB, HSD17B10, HS	1.52E-23
GO:0022627 ~ cytosolic small ribosomal subunit	RPS7, RPS6, RPSA, RPS4X, RPS15, RPS14, RPS17, RPS28, RPS15A, RPS16, RPS19, RPS29, RPS20, RPS2, FAU, RPS13, RPS24, RPS23	9.55E-24
mmu00020:Citrate cycle (TCA cycle)	CS, ACLY, PDHA1, PCX, IDH2, OGDH, PDHB, DLAT, SDHA, SDHB, DLD, IDH3A	4.39E-12
GO:0006086 ~ acetyl-CoA biosynthetic process from pyruvate	PDHA1, MPC1, MPC2, VDAC1, PDHB, DLAT, DLD	1.42E-10
mmu01230:Biosynthesis of amino acids	CS, TPI1, PCX, GOT1, PGAM1, IDH2, TKT, BCAT2, IDH3A	1.27E-04
KW-0249 ~ Electron transport	NDUFA8, NDUFA4, NDUFA10, UQCRC1, CYCS, ETFA, GLRX, UQCRC2, SDHA, SDHB	5.14E-07
GO:0042744 ~ hydrogen peroxide catabolic process	PRDX2, PRDX4, GPX1, HBB-B2, HBB-B1	7.60E-04
GO:0006739 ~ NADP metabolic process	PCX, IDH2, G6PDX, ME1, PGD	3.97E-05
GO:0006635 ~ fatty acid beta-oxidation	ECHS1, ACAA2, IVD, ECHDC1, ACADM, HADH, HSD17B10	3.93E-05
KW-0679 ~ Respiratory chain	NDUFA8, NDUFA4, NDUFA10, UQCRC1, CYCS, UQCRC2	5.79E-04
**0.99 abundance and 0.33 FSR**		
GO:0005794 ~ Golgi apparatus	PITPNB, SYAP1, GBF1, H2-K1, DLG1, GOLGA5, TBC1D4, MAN2A1, LMAN2, AP1S1, APOE, CD14, COPE, TMED5, H2-Q10, MAPK3	0.00221
GO:0005789 ~ endoplasmic reticulum membrane	SEC24A, SAR1B, LRBA, PITPNB, CCDC47, ALG5, SLC33A1, CYP4B1, ACSL4, SGPP1, LMF1, KTN1, MTDH, FADS2, DLG1, TMX2, RHEB, TMEM33, LMAN2, DHRS7B, IGFALS, ESYT1, FADS1, TMED5	6.84E-09
GO:0005777 ~ peroxisome	ABCD3, DHRS7B, GBF1, AGPS, ACSL4, FAR1	0.0062
GO:0003729 ~ mRNA binding	DDX3Y, RBM47, CNBP, KHSRP, STRAP, HNRNPA1, RBMX, EIF4G2, EIF2A, SF1	5.21E-04
GO:0005681 ~ spliceosomal complex	RHEB, U2AF2, PRPF19, HNRNPA1, RBMX, PRPF8, SF1	0.00148
KW-0508 ~ mRNA splicing	RBM47, U2AF2, KHSRP, STRAP, PRPF19, HNRNPA1, RBMX, PRPF8, SF1	0.01343
GO:0006956 ~ complement activation	C4B, CFH, CFI, CFB	0.03632
**0.99 abundance and 0.99 FSR**		
GO:0005739 ~ mitochondrion	NDUFA11, GSTP1, RAP1GDS1, COX5B, BPHL, TIMM50, MCEE, CHCHD3, AUH, CAPN1, TGM2, PDHX, OXSM, NDUFA2, PDE2A, GSR, DNAJC11, COQ9, HOGA1, COX7A2L, NDUFS8, CHDH, SARDH, ACBD3, SLC25A4, CRAT	2.87E-13
mmu00190:Oxidative phosphorylation	COX7A2L, NDUFS8, NDUFA11, NDUFA2, COX5B	0.02067
KW-0931 ~ ER-Golgi transport	SEC16B, SEC13, SEC23A, TMED2, ERGIC3	0.0019

### Impact of high fat diet on protein abundance and FSR in the liver

**Fig 4:**
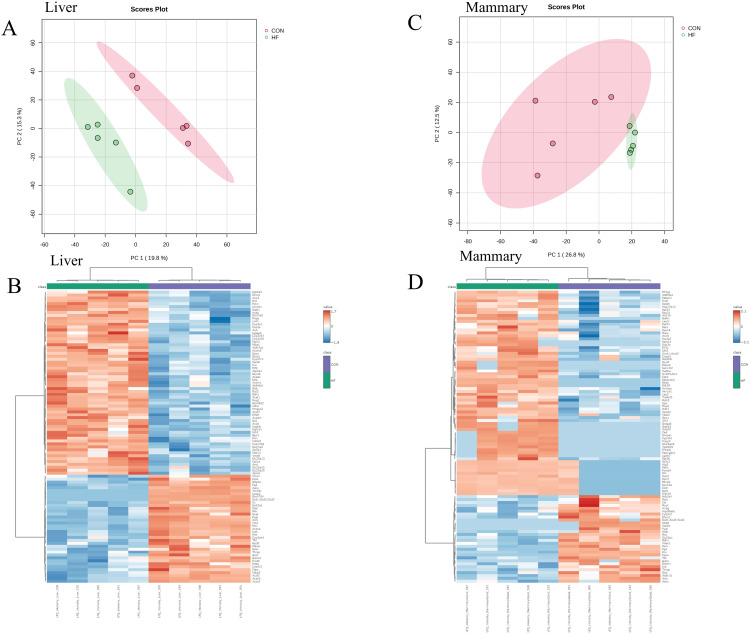
Principal component analysis (PCA) scores plot and hierarchical cluster analysis with a heatmap and dendrogram. 2A) PCA representing 1,830 liver proteins from mice fed a high fat (HF) or control (CON) diet demonstrated ~15 to ~19% of the variation can be accounted for by treatment. 2B) Heatmap with dendrogram representing hierarchical cluster analysis of the 100 most abundant proteins of liver demonstrated distinct clustering of the top most abundant proteins by treatment. 2C) PCA representing 1,261 mammary proteins from mice fed a HF or CON diet demonstrated ~12.5% of the variation in CON animals is due to individual animal, whereas ~27% of the variation is accounted for by treatment. 2D) Heatmap with dendrogram representing hierarchical cluster analysis of the top 100 proteins of mammary gland demonstrated distinct clustering of the top most abundant proteins by treatment.

**Fig 5:**
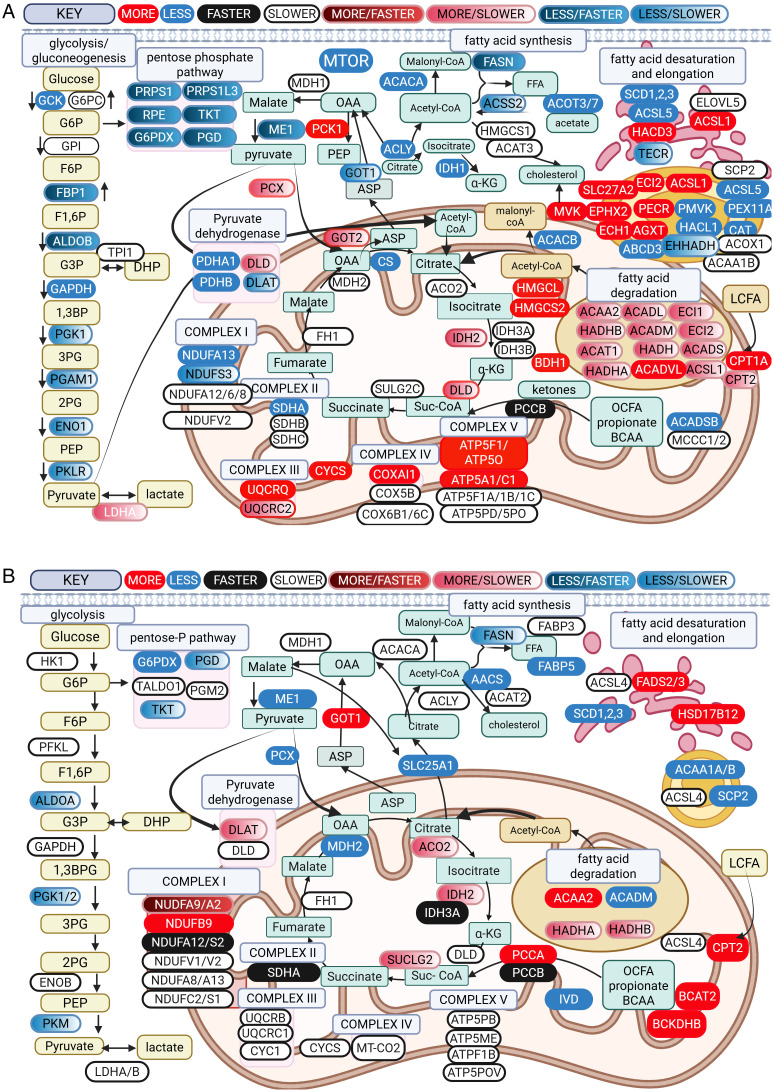
Schematic representative of proteins with differential abundance or different fractional synthetic rate (FSR) in liver (A) and mammary gland (B) of female ICR mice that consumed a high fat (HF) diet. Proteins with increased abundance are shown in red, decreased abundance are shown in blue, faster FSR is black, and slower FSR is white. Proteins that demonstrated greater abundance and faster FSR are shown as red/black boxes, greater abundance and slower FSR shown as red/white boxes, less abundance and faster FSR are blue/black, and less abundance and slower FSR are blue/white. Yellow boxes represent glycolytic intermediates, teal boxes represent citrate cycle and other metabolic intermediates, and tan boxes represent long chain fatty acid metabolism. Definitions of metabolite and protein abbreviations can be found in Appendix A in S2 Text for Fig 5A and Appendix B in S3 Text for Fig 5B. Reprinted from Biorender.com under a CC BY license, with permission from Biorender.com, original copyright 2026.

Multivariate analysis indicated that HF diet had a profound effect on liver protein abundance, with principal component analysis (PCA) scores plots and heat maps with dendrograms demonstrating distinct clustering of liver samples by HF and CON diet groups (Fig 4). T-test analysis found a total of 414 DAP in liver between HF and CON mice (raw P-value < 0.05), of these, 261 were greater in HF and 153 were less abundant. Almost half (47%; 117) of the proteins greater in abundance in the liver in response to HF diet were mitochondrial proteins that mediate fatty acid transport into the mitochondria (CPT1A, CPT2), breakdown of fatty acids via β-oxidation, as well as enzymes that mediate the synthesis of ketones (HMGCL, HMGCS2, BDH1), and represent components of complex III, IV and IV of the electron transport chain (Table 4 and Fig 5). Enzymes that regulate citrate cycle reactions (IDH2, DLD), conversion of oxaloacetate (OAA) to aspartate in the mitochondria (GOT2), carboxylation of pyruvate into mitochondrial OAA (PCX), and conversion of cytosolic OAA into phosphoenolpyruvate (PEP) via cytosolic phosphoenolpyruvate carboxykinase (PCK1) in the cytosol were greater in abundance in liver of HF mice (Table 4 and Fig 5). Also increased in abundance were peroxisomal proteins, proteins that mediate xenobiotic metabolic processes, multiple apolipoproteins (APOA1BP, APOA4, APOL), and multiple UDP-glucuronosyltransferase enzymes that facilitate the elimination of various toxic substances (UGT2B34, UGT1A1, UGT2B1, UGT3A2, UGT2A3, UGT1A6B).

**Table 4:** Categories enriched by liver proteins with differential abundance or differential fractional synthetic rate (FSR) between mice fed a high fat (HF) diet or control (CON) diet.

Term	Genes	FDR
**More abundant in liver of HF mice**		
GO:0005739 ~ mitochondrion	PECR, ACAA2, ISOC2A, GLDC, ECI1, ACSM1, ECI2, ACSM5, GHITM, CYP2D10, MPC2, SLC25A42, HADH, ACAD10, CPT1A, ACSL1, ECH1, HOGA1, KMO, HDHD3, CYP2D26, VDAC3, CYP2E1, UQCRC2, SLC27A2, ALDH7A1, ATP6V1A, MAOB, ABCB7, AK4, GLYAT, ACAT1, LDHA, DECR1, APOOL, AGXT2, IDH2, GOT2, IRGM1, RMDN2, MCAT, ACSF2, GLUD1, ALDH6A1, UQCRQ, CYCS, ECHDC2, ECHDC3, NIT1, NIT2, ACADVL, ABCD2, HIBADH, COX4I1, ETFA, ETFB, AIFM1, CPT2, ACADL, AUH, NNT, DBT, HMGCS2, ACADM, ACADS, ACAD8, HSDL2, GPX1, AMT, SORD, SIRT3, VWA8, HADHB, HADHA, SLC25A15, ALDH5A1, BDH1, GM4952, IVD, CHDH, CANX, AGXT, MRRF, DLD, SLC25A13, ECHS1, PCX, ETFDH, DLST, DDX21, MRPS30, HSD17B10, HSD17B8, BPHL, NT5C, GULO, HMGCL, SAMM50, ALDH1B1, IARS2, SLC25A20, SLC25A22, CBR4, BCAP31, CYB5A, FAHD1, GK, AADAT, GLYCTK, SUOX, DHRS4, ETNPPL, AGMAT, ALDH4A1, XPNPEP3, SARDH, OTC	1.86E-69
GO:0006629 ~ lipid metabolic process	PECR, ACADVL, ABCD2, ACAA2, ACSM1, ECI1, ACSM5, CYP2C39, CYP2C37, CPT2, ACADL, AOX1, HMGCS2, ACADM, HADH, ACADS, ACAD8, CPT1A, GPX1, PCYT2, UGT1A1, ACSL1, SACM1L, ECH1, HADHB, CYP39A1, HADHA, BDH1, CYP2E1, SLC27A2, PAFAH1B2, SLC27A5, ECHS1, MVK, PCX, HSD17B13, HSD17B6, HACD3, HSD17B8, HSD17B10, CYP7A1, ACAT1, C3, ADH4, HMGCL, PCK1, CBR4, DECR1, EPHX2, EPHX1, NAGA, FMO5, MCAT, AKR1C6, ACSF2, CYP1A2, ECHDC2, ECHDC3	9.83E-36
GO:0006635 ~ fatty acid beta-oxidation	ACAD8, ACADVL, ABCD2, CPT1A, ECHS1, ACAA2, ECI1, ECI2, ECH1, HSD17B10, ACAT1, HADHB, HADHA, CPT2, AUH, IVD, ECHDC2, ACADM, HADH, SLC27A2, DECR1	1.79E-25
GO:0005783 ~ endoplasmic reticulum	UGT2B1, RDH7, ATP2A2, CYP2C39, CES3A, CYP2C37, SERPINA1D, CYP2D10, CYP2B10, CLCC1, COLGALT1, PDIA3, UGT1A1, SSR4, ACSL1, SACM1L, ATP11C, MOGS, DDOST, PDIA5, UGT1A6B, PDIA4, CYP2D26, HADHB, CYP39A1, DNAJC3, POR, CANX, CYP2E1, SLC27A2, ERGIC1, SLC27A5, H6PD, RPN1, HSD17B13, HSD17B6, HACD3, HSD17B10, CYP7A1, HSP90B1, LMF1, ACAT1, CYP2B9, GULO, GANAB, CYP2C54, CYP2C50, RPL18, PCK1, UGGT1, SEC11A, TXNDC5, BCAP31, CYB5A, ERAP1, EPHX1, FMO1, IRGM1, FMO3, FMO5, CYP4F14, KTN1, RAB10, GLUD1, CYP1A2, HYOU1, P4HB	3.26E-25
GO:0006805 ~ xenobiotic metabolic process	FMO1, FMO3, FMO5, UGT1A6B, CYP2C39, CYP2D26, CYP2B9, CYP2C37, CYP2B13, CYP2D10, CYP2C54, CYP2B10, AOX3, CYP1A2, CYP2C50, AOX1, CYP2E1	1.30E-13
GO:0033539 ~ fatty acid beta-oxidation using acyl-CoA dehydrogenase	ACADVL, ACADL, IVD, ETFDH, ETFA, ACADM, ETFB, ACAD10, ACADS	5.55E-12
GO:0016712 ~ oxidoreductase activity, acting on paired donors, with incorporation or reduction of molecular oxygen, reduced flavin or flavoprotein as one donor, and incorporation of one atom of oxygen	CYP2B9, CYP2C37, CYP2B13, CYP2D10, CYP2C54, CYP2B10, CYP1A2, CYP2C50, CYP2E1, CYP2C39, CYP2D26	5.69E-09
GO:0042178 ~ xenobiotic catabolic process	CYP2B9, CYP2B13, ABCC2, ACSL1, CYP2B10, CYP1A2	0.0001619
GO:0005777 ~ peroxisome	PECR, ABCD2, HSDL2, MVK, ACSL1, EPHX2, ECI2, ECH1, IDH2, DHRS4, VWA8, HMGCL, LONP2, AGXT, SLC27A2	6.18E-10
mmu01210:2-Oxocarboxylic acid metabolism	AADAT, AGXT2, DBT, IDH2, GOT2, DLST, AGXT, DLD	3.39E-05
GO:0009055 ~ electron transfer activity	POR, AOX3, ETFDH, AOX1, CYCS, ETFA, ETFB	2.29E-06
mmu00980:Metabolism of xenobiotics by cytochrome P450	ADH4, UGT2B34, UGT1A1, UGT2B1, CYP1A2, EPHX1, UGT2A3, CYP2E1, UGT1A6B	0.000823
mmu04976:Bile secretion	UGT2B34, ABCC2, UGT1A1, UGT2B1, ABCB4, EPHX1, UGT2A3, CYP7A1, UGT1A6B, SLC27A5	0.0010925
GO:0001676 ~ long-chain fatty acid metabolic process	CPT1A, CPT2, ACSL1, CYP2E1, SLC27A2, SLC27A5	0.0001619
mmu03320:PPAR signaling pathway	CPT1A, CPT2, ACADL, GK, ACSL1, HMGCS2, ACADM, PCK1, CYP7A1, SLC27A2, SLC27A5	7.92E-05
GO:0016746 ~ acyltransferase activity	HADHB, CPT1A, ACAA2, CPT2, GM4952, DBT, DLST, HMGCS2, GLYAT, ACAT1	0.0002712
GO:0006633 ~ fatty acid biosynthetic process	PECR, ACSM1, ACSM5, HACD3, HSD17B8, MCAT, CBR4	0.0022646
GO:0050661 ~ NADP binding	GRHPR, POR, H6PD, HIBADH, NNT, FMO1, FMO3, FMO5	1.92E-06
GO:0009055 ~ electron transfer activity	POR, AOX3, ETFDH, AOX1, CYCS, ETFA, ETFB	2.29E-06
GO:0042626 ~ ATPase-coupled transmembrane transporter activity	ABCD2, ABCC2, ABCB7, ABCB4, ABCC6	0.0052003
GO:0006457 ~ protein folding	PDIA3, CANX, P4HB, PDIA5, PDIA4, HSP90B1, TXNDC5	0.0170808
**Less abundant in liver of HF mice**		
GO:0006629 ~ lipid metabolic process	ACSS2, TECR, MTTP, ABHD5, ACACB, ACACA, AACS, CYP17A1, CYB5R3, RDH11, PMVK, SCD2, RDH16, SCD3, HACL1, SCD1, FDPS, GSTM2, ACOT7, ACSL5, ACADSB, ACLY, GPAM, FABP5, FASN, EHHADH, ASPG, THRSP, ACOT3	1.06E-13
GO:0006633 ~ fatty acid biosynthetic process	ACLY, ABCD3, FASN, TECR, SCD2, SCD3, SCD1, ACACB, ACACA	7.24E-07
GO:0006084 ~ acetyl-CoA metabolic process	CS, ACLY, FASN, ACACB, ACACA	1.42E-05
GO:0008610 ~ lipid biosynthetic process	ACLY, ACSS2, FASN, SCD1, ACACA	0.0005578
mmu00010:Glycolysis/ Gluconeogenesis	PDHA1, ACSS2, PKLR, PGAM1, PDHB, ENO1, GCK, ALDH2, PGK1, DLAT, ALDOB, GAPDH, FBP1	1.36E-09
mmu04922:Glucagon signaling pathway	PDHA1, PGAM1, PDHB, PYGL, ACACB, FBP1, GCK, ACACA	0.0037572
mmu04910:Insulin signaling pathway	PKLR, FASN, PYGL, ACACB, FBP1, GCK, ACACA, MTOR	0.017069
mmu00030:Pentose phosphate pathway	PRPS1, RPE, G6PDX, ALDOB, PGD, TKT, FBP1, PRPS1L3	4.24E-06
GO:0005783 ~ endoplasmic reticulum	RPL10, CNBP, TECR, RPL10L, MTTP, ACSL5, FMO4, A1CF, MTOR, CYP17A1, EEF1G, SYNCRIP, PGRMC2, NPLOC4, CYB5R3, CES2E, CTTN, RDH11, RDH16, UGT1A5, SCD2, SCD3, SCD1, SEC23B	0.0003195
GO:0005777 ~ peroxisome	FDPS, MAVS, ABCD3, EHHADH, IDH1, PEX11A, CAT, HACL1, TKT, ACOT3	1.82E-06
GO:0006099 ~ tricarboxylic acid cycle	CS, ACLY, PDHA1, IDH1, PDHB, DLAT, SDHA	3.22E-06
mmu01040:Biosynthesis of unsaturated fatty acids	ACOT7, TECR, SCD2, SCD3, SCD1, ACOT3	0.0010369
GO:0032869 ~ cellular response to insulin stimulus	PKLR, GPAM, GOT1, FBP1, GCK, MTOR	0.0104511
GO:0008202 ~ steroid metabolic process	FDPS, CYB5R3, MTTP, AKR1C13, PMVK, CYP3A44, RDH16, CYP17A1	0.0004144
**Faster FSR in liver of HF mice**		
GO:0005737 ~ cytoplasm	CLIC4, NDUFA13, ACY1, DPYS, NGLY1, YARS1, CLU, RPL6, ACTG1, PPP6C, SYNCRIP, AS3MT, FTL1, LGALS1, OPA1, NARS1, FTH1, ME1, KIF13B, VPS35, PGLS, GLUL, GSTO1, PGAM1, ANXA5, MARS1, AKR1A1, EPS15L1, PTGR2, SND1, ACLY, PPA1, EEF1D, AKR1C14, TMBIM6, LCP1, LAP3, HPRT1, KPNB1, SEC23A, AARS2, DHX9, RAP1GDS1, AK3, PRDM11, NT5C, BCLAF1, EPS8L2, PCBD1, SULT3A2, CAR3, HARS1, GSTM1, SEPTIN2, EPHX2, IDH1, AGL, TXNL1, RMDN3, PYCR3, IIGP1, SEPTIN7, ARCN1, HAL, PFKL, FASN, KYAT3, OGT, FARSB	7.29E-16
GO:0005783 ~ endoplasmic reticulum	SEC23A, RPN2, ATL3, CAV1, RAP1GDS1, ATP2A2, CLU, CYP3A16, RPL6, PDIA5, IIGP1, ARCN1, SEC61A1, SYNCRIP, NCLN, SPCS1, EEF1D, ILVBL, CYP1A2, RAB18, TMBIM6, GUSB, GLUL	5.64E-06
GO:0016491 ~ oxidoreductase activity	MAOB, GSTO1, IDH1, ETFDH, AKR1A1, PYCR3, PTGR2, CYP3A16, ALDH1B1, IVD, FTH1, FASN, CYP1A2, ME1, ALDH16A1	4.16E-06
mmu01230:Biosynthesis of amino acids	PFKL, ACY1, IDH1, PGAM1, PYCR3, TKT, GLUL	0.0014676
GO:0042178 ~ xenobiotic catabolic process	GSTM1, GSTO1, CYP1A2, CYP3A16	0.0704017
mmu00030:Pentose phosphate pathway	PFKL, PGLS, TKT	0.1751108
GO:0016829 ~ lyase activity	CAR3, HAL, ILVBL, FASN, CYP1A2, KYAT3, PCBD1	0.0012155
**Slower FSR in liver of HF mice**		
mmu00591:Linoleic acid metabolism	CYP2C37, CYP2C69, CYP2C54, CYP3A41B, CYP2C40, CYP2C29, CYP2C39, CYP2C38	0.0232056
GO:0006531 ~ aspartate metabolic process	GOT1, GOT2, ADSS2, ASS1	0.0073917
GO:0006096 ~ glycolytic process	GPI1, TPI1, PKLR, ENO1B, PGK1, ALDOB, DHTKD1	0.0058783
GO:0006520 ~ amino acid metabolic process	PM20D1, GLUD1, GOT1, GLDC, GOT2, ASL	0.0022661
mmu00980:Metabolism of xenobiotics by cytochrome P450	GSTK1, CBR1, ADH1, UGT1A1, UGT2B1, GSTA4, GSTP2, GSTA3, EPHX1, GSTT2, UGT2A3, CYP2F2	0.0008536
GO:0006886 ~ intracellular protein transport	RAB2A, NAPA, COPA, RAB7, TMED10, RAB5C, PDCD6, COPB1, CSE1L, USO1, CLTA, AP2A1, AP2B1, EHD1, COPG1, ARF5, SEC31A	0.000789
mmu00190:Oxidative phosphorylation	NDUFA8, NDUFA6, ATP5PD, NDUFA12, SDHC, COX6C, ATP5F1C, COX5B, SDHB, COX6B1, ATP5F1A, ATP5F1B, ATP5PO, NDUFS3, NDUFS2, UQCRC2, NDUFV2	0.0006329
mmu00220:Arginine biosynthesis	GLUD1, GOT1, ARG1, GLS2, GOT2, ASL, ASS1	0.0006197
mmu00590:Arachidonic acid metabolism	CBR1, GPX4, HPGD, CYP2C29, CYP2C39, CYP2C38, CYP2B9, CYP2C37, CYP2C69, CYP2B13, CYP2C54, CYP2B10, CYP2C40, LTA4H	0.0002283
mmu00250:Alanine, aspartate and glutamate metabolism	GLUD1, ALDH5A1, GOT1, AGXT2, GLS2, GOT2, ABAT, ASL, ADSS2, ASS1	0.0001133
GO:0003988 ~ acetyl-CoA C-acyltransferase activity	HADHB, HADHA, ACAA2, SCP2, ACAA1B	5.33E-05
KW-0648 ~ Protein biosynthesis	EIF5A, EIF4A2, EIF4A1, EIF2B2, KARS1, TUFM, EEF1A1, SARS1, EIF3M, LARS1, IARS2, IARS1, CARS1, EIF4E, EIF4G2, VARS1	3.18E-05
GO:0000062 ~ fatty-acyl-CoA binding	HADHA, ACADL, SCP2, ECI2, DBI, ACADM, ACADS	3.07E-05
GO:0000302 ~ response to reactive oxygen species	ALDH3A2, GPX1, GSTP2, PRDX1, CAT, SOD2, PRDX6, SOD1	1.61E-05
MOTIF:Prevents secretion from ER	PDIA3, HSPA5, G6PC1, PDIA6, HSP90B1, CES3B, ALDH3A2, NSDHL, CES1D, CES1F, P4HB, CALR, PPIB	3.88E-06
mmu00020:Citrate cycle (TCA cycle)	PCX, MDH1, MDH2, IDH2, IDH3B, SUCLG2, SDHC, ACO2, SDHB, DLD, IDH3A	2.25E-06
mmu00010:Glycolysis/ Gluconeogenesis	TPI1, ACSS2, PKLR, ENO1B, G6PC1, ALDH3A2, GPI1, ADH1, LDHA, ALDH2, PGK1, ALDOB, DLD, ALDH7A1, FBP1, ALDH9A1	4.25E-07
DOMAIN:Aldehyde dehydrogenase	ALDH3A2, ALDH1L1, ALDH6A1, ALDH5A1, ALDH2, ALDH1A1, ALDH7A1, ALDH1A7, ALDH9A1	1.75E-07
GO:0006457 ~ protein folding	PDIA3, CCT3, HSPA8, HSP90AA1, HSP90AB1, AHSA1, HSP90B1, HSPD1, DNAJA1, CCT6A, HSPH1, GRPEL1, CANX, P4HB, CALR, PPIB, PPIA, CCT4	2.14E-09
mmu04146:Peroxisome	PEX16, GSTK1, PECR, ABCD3, ACSL1, PEX11A, ECI2, IDH2, HSD17B4, PIPOX, ACAA1B, ACNAT2, SOD2, DHRS4, SOD1, PRDX5, SCP2, ACOX1, EHHADH, PRDX1, PXMP2, CAT, DECR2	4.18E-11
GO:0006979 ~ response to oxidative stress	MAP2K1, GPX1, NDUFA6, GPX4, 1600014C10RIK, NDUFA12, PARK7, COMT, SOD2, PRDX6, SOD1, ALAD, PRDX3, PRDX5, PRDX4, PSMB5, PRDX1, CAT, G6PDX, ALDH1A1, NDUFS2, APOE	1.22E-11
GO:0008203 ~ cholesterol metabolic process	FDPS, HMGCS1, PON1, HDLBP, APOA4, CYP51, CYP27A1, LIMA1, NSDHL, EBP, CYB5R3, CES1D, CES1F, FDX1, CAT, CES1G, MVD, HMGCS2, APOE, APOB, SULT2A2	8.74E-12
mmu01212:Fatty acid metabolism	CPT1A, ECHS1, ACAA2, ELOVL5, ACSL1, TECR, HSD17B4, ACAA1B, HACD3, HSD17B8, ACAT3, ACAT1, HADHB, HADHA, CPT2, ACADL, SCP2, ACOX1, EHHADH, ACADM, HADH, ACADS	3.03E-13
mmu00071:Fatty acid degradation	CPT1A, ECHS1, ACAA2, ACSL1, ECI1, ECI2, ACAA1B, ACAT3, ACAT1, HADHB, ALDH3A2, HADHA, ADH1, CPT2, ACADL, ALDH2, ACOX1, EHHADH, ACADM, HADH, ALDH7A1, ACADS, ALDH9A1	2.90E-16
mmu00280:Valine, leucine and isoleucine degradation	ECHS1, ACAA2, HIBADH, ACAA1B, ABAT, ACAT3, ACAT1, ALDH2, HMGCS2, ACADM, HADH, ACADS, HIBCH, MCCC2, HMGCS1, AGXT2, MCCC1, ALDH3A2, HADHB, HADHA, ALDH6A1, EHHADH, DLD, ALDH7A1, ALDH9A1	1.31E-17
GO:0006635 ~ fatty acid beta-oxidation	CPT1A, ABCD3, ECHS1, ACAA2, ECI1, ECI2, HSD17B4, ACAA1B, ACAT3, ACAT1, HADHB, HADHA, CPT2, SCP2, ACOX1, EHHADH, ECHDC2, ACADM, HADH, DECR1	1.02E-17
GO:0005777 ~ peroxisome	PECR, ABCD3, PEX11A, ECI2, HSD17B4, ACAA1B, ACNAT2, PRDX5, SCP2, ISOC1, DECR2, PEX16, ATAD1, GSTK1, FDPS, ACSL1, IDH2, PIPOX, DHRS4, SOD1, ALDH3A2, VWA8, TMEM135, ACOX1, EHHADH, CAT, PXMP2, MVD, UOX	2.85E-20
GO:0005789 ~ endoplasmic reticulum membrane	TRAM1, ARL6IP1, PDCD6, TECR, CYP2C39, CYP2C38, CYP2C37, NSDHL, CYP2B13, CYP2D10, CYP2B10, NAT8F2, BSG, ANXA7, CLCC1, RAB2A, EIF5A, PCYT1A, UGT1A1, SSR4, ACSL1, ELOVL5, DNAJB12, ATP11C, DDOST, TMT1B, CYP2D26, ALDH3A2, POR, CYP2D22, CYP2A5, TMX2, CANX, CYP3A41B, CYP2C40, PLPP3, SEC22B, HPD, SLC27A5, CERS2, RTN3, TMED10, RRBP1, HACD3, HSP90B1, CYP17A1, LMF1, CYP2B9, GULO, LMAN1, EBP, PGRMC1, CYB5R3, CYP2A12, CYP2C54, SSR1, EMC6, CYP2F2, SEC31A, PEX16, CYB5A, HSPA5, EPHX1, FMO1, FMO3, CYP2C29, G6PC1, FMO5, CYP8B1, CYP4F14, CYP51, GGCX, STT3A, CALR	7.82E-23
GO:0006412 ~ translation	RPL4, EIF4A2, RPL5, EIF4A1, RPL3, KARS1, MRPL39, RPL8, MRPL12, RPL9, RPL7, RPS14, RPS17, RPS19, RPS3, RPL13, IARS2, RPS2, RPL15, IARS1, RPS11, RPS27A, EIF4E, RPS13, VARS1, EIF5A, EIF2B2, RPS7, RPL21, RPS5, RPL23, RPSA, TUFM, EEF1A1, SARS1, EIF3M, LARS1, GM6436, RPS20, RPL27, RPL22L1, FAU, CARS1, RPS24, EIF4G2, RPS23	2.10E-24
GO:0022626 ~ cytosolic ribosome	RPL4, RPL5, RPL3, RPLP0, RPL8, RPL9, RPL7, RPS14, RPL7A, RPS17, RPS19, RPS3, RPL13, RPS2, RPL15, RPS11, RPS27A, RPS13, GCN1, RPS7, RPL21, RPS5, RPL23, RPSA, EEF1A1, RPS20, RPL27, FAU, ABCE1, RPS24, RPS23	1.30E-25
GO:0005783 ~ endoplasmic reticulum	RPL5, ARL6IP1, PDCD6, PARK7, NSDHL, CYP2D10, SCP2, BSG, CLCC1, TGM2, RAB2A, PDIA3, MAP2K1, RPS7, RPL21, ACSL1, DNAJB12, ATP11C, PDIA6, CYP2D26, ALDH3A2, POR, CYP2D22, TMX2, LARS1, RPL27, PLPP3, PPIB, HPD, SLC27A5, RTN3, TMED10, HACD3, HSP90B1, ACAT1, CYP2B9, VTN, EBP, PRDX4, CYB5R3, SSR1, APOE, APOB, SEC31A, EIF2B2, HSPA5, EPHX1, CYP2C29, G6PC1, CYP51, CYP4F14, GLUD1, GGCX, STT3A, CALR, RPS24, RPS23, TRAM1, PIGT, UGT2B1, TECR, RPLP0, DBI, YBX1, CYP2C39, CYP2C38, CYP2C37, CES2A, SERPINA1B, CD1D1, SERPINA1C, CES2E, CYP2B10, NAT8F2, EIF5A, PCYT1A, SSR4, UGT1A1, ELOVL5, TMT1A, AHSA1, DDOST, TMT1B, CES3B, HADHB, CYP2A5, CANX, CYP3A41B, CYP2C40, SEC22B, CERS2, AHCY, USO1, RRBP1, HSD17B6, ATP1A1, HSD17B11, CYP17A1, LMF1, GULO, LMAN1, GANAB, PGRMC1, SRP72, CYP2A12, CYP2C54, RPS3, RPL13, EMC6, CYP2F2, PEX16, PTPN1, CYB5A, 1600014C10RIK, FMO1, FMO3, DHRS1, CYP8B1, FMO5, DNAJA1, CYP2C69, UFM1, CES1D, CES1F, CES1G, P4HB, COPG1	8.79E-45
GO:0006629 ~ lipid metabolic process	PECR, RAB7, ACAA2, ACSM3, ACSM1, ECI1, TECR, PRKAG1, HDLBP, ACSM5, COMT, CYP2C39, CYP2C38, CYP2C37, NSDHL, SCP2, CPT2, ACADL, HMGCS2, ACADM, HADH, ACADS, SULT2A2, CBR1, CPT1A, GPX1, PCYT1A, UGT1A1, HMGCS1, GPX4, ACSL1, ELOVL5, ALDH3A2, CYP27A1, HADHB, HADHA, SULT1D1, BDH1, ACOX1, EHHADH, ALDH1A1, FDX1, CYP2C40, ACOT1, MVD, PLPP3, SLC27A5, PAFAH1B1, CERS2, ECHS1, ACSS2, PCX, HPGD, AKR1D1, HSD17B4, ACAA1B, HSD17B6, HACD3, ACNAT2, HSD17B11, HSD17B8, CYP17A1, ACAT1, SULT1A1, C3, LIMA1, EBP, ATP5F1A, ATP5F1B, CYB5R3, APOE, DECR2, APOB, DECR1, FDPS, EPHX1, FAH, CYP2C29, FMO5, CYP8B1, PRDX6, AKR1C6, ACSF2, CYP51, PM20D1, GPAM, FABP5, CES1D, GSTA3, CES1G, ECHDC2	4.72E-47
GO:0005739 ~ mitochondrion	DMGDH, PHB1, ACSM3, GLDC, ECI1, ACSM1, ECI2, ACSM5, ABAT, PHB2, RPS14, SCP2, DPYSL2, LONP1, ACAD10, PDK1, MCCC2, GSTK1, CPT1A, TSTD1, ACSL1, MCCC1, CYP27A1, MTHFD1, LACTB2, FDX1, ACOT1, SUCLG2, UQCRC2, ALDH7A1, MRPL12, PRDX3, ADH1, PRDX5, LDHA, PRDX4, CYB5R3, GRPEL1, PRDX1, LDHD, DECR1, CTSA, APOOL, PRDX6, MSRA, GLUD1, ALDH6A1, PRXL2A, ECHDC2, SFXN1, RAB7, HIBADH, MRPL39, ACADL, NNT, ACADM, ACADS, AASS, CTSB, FAHD2A, VWA8, HADHB, HADHA, ALDH5A1, BDH1, GM4952, CANX, DLD, ECHS1, PCX, GSTP2, KARS1, HSD17B4, COX5B, HSD17B8, CYP17A1, GULO, PGRMC1, ATP5F1A, SAMM50, ATP5F1B, RPS3, ATAD1, ATP5PD, MDH1, 1600014C10RIK, GK, MDH2, GLYCTK, SQOR, COX6C, ATP5F1C, DHRS1, LRPPRC, DHRS4, AGMAT, GPAM, ATP5PO, UOX, PECR, MTCH2, ALDH1L1, ACAA2, NDUFA12, CPOX, PARK7, COMT, CYP2D10, KIF5B, HADH, TMEM14C, TGM2, MAP2K1, SDHC, KMO, SDHB, COX6B1, TUFM, CYP2D26, ALDH3A2, TMEM135, CYP2D22, TMX2, FKBP4, AK2, AK4, GLYAT, ACAT3, ACAT1, NDUFV2, VARS1, FDPS, HSPA5, AGXT2, IDH2, GOT2, COQ6, ACSF2, QDPR, PXMP2, ACO2, ADSS2, SLC25A1, HSP90AB1, ABCD3, ETFA, DBI, ETFB, DHTKD1, HEBP1, NADK2, CPT2, ALDH2, IDH3B, HMGCS2, HIBCH, IDH3A, HSP90AA1, GPX1, GPX4, NIPSNAP1, SARS1, DDAH1, TST, ACOX1, CHDH, EHHADH, CAT, NDUFS3, NDUFS2, SLC25A10, SLC25A5, SLC25A4, SLC25A13, ALDH9A1, NAXE, GLS2, ACAA1B, BPHL, HSPD1, IARS2, CYB5A, NDUFA8, FAHD1, NDUFA6, SOD2, SUOX, ASS1, SOD1, GSTZ1, DNAJA1, SARDH, OCIAD1, SLC25A32, ABCE1	8.56E-98

Proteins less abundant in liver of HF diet mice included glycolytic/gluconeogenic enzymes, enzymes within the pentose phosphate pathway, components of the pyruvate dehydrogenase enzyme (DLAT, PDHA1, PDHB), proteins of complex I and II of the electron transport chain, and enzymes that mediate *de novo* fatty acid (FASN) synthesis and mono-unsaturation (SCD1, SCD2, SCD3) as well as the mammalian target of rapamycin (mTOR) protein, which is central in many cellular processes like protein synthesis.

There were 621 liver proteins with differential FSR between HF and CON mice (raw P-value < 0.05), of these proteins, 109 had faster FSR and 512 (82.4%) slower FSR in HF animals. Proteins with faster FSR included glycolytic/gluconeogenic enzymes (ALDOB, FBP1), multiple enzymes in the pentose phosphate pathway, malic enzyme 1 (ME1), fatty acid synthase (FASN) and the beta subunit of the propionyl-CoA carboxylase enzyme (PCCB) (Table 4 and Fig 5). Liver proteins with slower FSR in HF mice included most enzymes that mediate citrate cycle reactions, proteins within complex I and II of the electron transport chain, and multiple enzymes that mediate fatty acid degradation in the mitochondria.

### Impact of high fat diet on protein abundance and FSR in the mammary gland

Scores plot of PCA of the mammary proteome showed distinct separation of samples between HF and CON diets. Heat maps with dendrogram to visualize hierarchical clusters of the top 100 differential proteins also showed clear distinct clustering of proteins by abundance between the different diets (Fig 4). T-test analysis comparing LFQ of proteins of HF and CON groups found 254 DAP in the mammary gland (raw P-value < 0.05). Of the 254 DAP, 199 were greater in HF and 55 were less in HF than CON. Proteins greater in abundance in the mammary gland of HF mice included large and small ribosomal subunits, enzymes that mediate mitochondrial fatty acid β-oxidation (ACAA2, HADHA, HADHB), fatty acid elongation (HSD17B12) and desaturation (FADS2 and FADS3) in the endoplasmic reticulum, and the breakdown of branched chain amino acids (BCAT2, BCKDHB) as well as several citrate cycle enzymes (ACO2, IDH2, SUCLG2; Table 5 and Fig 5). Proteins lower in abundance in mammary glands of HF diet mice function as enzymes in glycolysis and the pentose phosphate pathway as well as FASN and SCD1, SCD2, and SCD3.

**Table 5:** Categories enriched by mammary gland proteins with differential abundance or differential fractional synthetic rate (FSR) between mice fed a high fat (HF) diet or control (CON) diet.

Category	Genes	FDR
**More abundant in mammary gland of HF mice**		
GO:0003735 ~ structural constituent of ribosome	RPL30, RPS9, GM49804, RPL23, RPS6, RPL11, RPL13A, RPL9, RPS15, RPS15A, RPS16, UBB, RPL36, RPS3, RPL24, KXD1, RPL13, RPL27, RPS27A, UBA52, RPS21	3.4E-14
GO:0015031 ~ protein transport	ARF4, RAB1A, TRAM1, SEC23A, TMED10, CSE1L, AP3D1, TIMM13, COG1, RRBP1, KTN1, SEC61A1, AP1G2, VPS35, PREB, SEC22B, AP2M1, SEC63, AP1M1, ARL8B	3.7E-06
GO:0006629 ~ lipid metabolic process	GPX1, ACAA2, ACSL1, OXSM, BCKDHB, HDLBP, AKR1A1, PTPN11, HSD17B12, AGPAT1, HSD17B10, HADHB, FADS2, HMGCL, HADHA, CPT2, PCCA, ILVBL, ACOT2, ACOT1, ACBD3, PAFAH1B3, FADS1, BCAT2	6.1E-08
GO:0005783 ~ endoplasmic reticulum	RAB1A, TRAM1, SEC23A, TMED10, CCDC47, PITPNB, RAP1GDS1, RRBP1, ITPR3, HSD17B12, AGPAT1, HSD17B10, MTDH, FADS2, SEC61A1, DDRGK1, RPS3, RPL13, GGT1, UGGT1, FADS1, SEC63, TXNDC5, PTPN1, MAP2K1, ACSL1, RPS6, ALG5, SLC33A1, PDE2A, CYP4B1, CNPY2, ATP2B2, KTN1, TMCO1, MANF, HADHB, DLG1, NPLOC4, REEP5, ILVBL, SRPRB, RPL24, PREB, RPL27, SEC22B, UBA52, RPS21	5.5E-17
IPR019956:Ubiquitin_dom	UBB, UBC, KXD1, GM8797, RPS27A, UBA52	8.3E-05
GO:0005794 ~ Golgi apparatus	ARF4, RAB1A, ST6GAL1, MAP2K1, TMED10, GALNT3, PITPNB, SYAP1, PDE2A, AP3D1, COG1, TMCO1, DLG1, GOLGA5, CAND1, AP1G2, SRPRB, CD36, ACBD3, SEC22B, SCYL2, AP1M1	2.2E-03
GO:0006413 ~ translational initiation	EIF2B5, LARP1, EIF5, EIF3C, ABCE1, EIF3A, EIF4G1	5.7E-04
GO:0006099 ~ tricarboxylic acid cycle	IDH2, SUCLG2, ACO2, DLAT	1.0E-01
GO:0016746 ~ acyltransferase activity	HADHB, PDHX, ACAA2, CPT2, OXSM, DLAT, GGT1, AGPAT1	5.0E-03
GO:0008483 ~ transaminase activity	OAT, GOT1, GFPT1, BCAT2	1.5E-02
**Less abundant in mammary gland of HF mice**		
GO:0016740 ~ transferase activity	GSTM1, ACAA1B, ACAA1A, PYGL, PRDX6, GSTZ1, ACLY, PKM, SCP2, FASN, NAMPT, AGPS, PGK1, TKT, GLUL, GNE	5.1E-05
KW-0809 ~ Transit peptide	SLC25A1, PCX, BDH1, AIFM1, MDH2, IVD, AGPS, CPOX, ECHDC2, ACAA1B, ACADM, ACAA1A	1.3E-08
KW-0496 ~ Mitochondrion	SLC25A1, PCX, MDH2, NDUFA4, CPOX, CYB5R3, BDH1, AIFM1, SCP2, IVD, SFXN3, ECHDC2, VDAC1, ACADM, GLUL	1.5E-04
GO:0006629 ~ lipid metabolic process	OLAH, GSTM1, PCX, ACAA1B, CEL, ACAA1A, PRDX6, AACS, ACLY, CYB5R3, BDH1, FABP5, SCP2, FASN, AGPS, SCD2, SCD3, ECHDC2, THRSP, FAR1, ACADM, SCD1	5.5E-17
GO:0006739 ~ NADP metabolic process	PCX, G6PDX, ME1, PGD	1.2E-04
GO:0006635 ~ fatty acid beta-oxidation	SCP2, IVD, ECHDC2, ACAA1B, ACADM, ACAA1A	7.8E-06
GO:0046166 ~ glyceraldehyde-3-phosphate biosynthetic process	PCX, MDH2, PGK1, TKT	1.1E-04
GO:0006096 ~ glycolytic process	ALDOART1, PKM, PGK1, ALDOA	4.2E-03
GO:0006098 ~ pentose-phosphate shunt	G6PDX, PGD, TKT	7.6E-03
GO:0031966 ~ mitochondrial membrane	CYB5R3, NDUFA4, IVD, SFXN3, VDAC1, ACADM	5.4E-04
**Faster FSR in mammary gland of HF mice**		
GO:1990904 ~ ribonucleoprotein complex	RPL7A, RPS14, RPS9, RPS16, RPL18A, DHX9, HNRNPD, RPL13, RPS13, RPL7, ACTG1	1.2E-06
GO:0000166 ~ nucleotide binding	EIF4A2, DYNC1H1, DHX9, UBA5, TUBB4B, AACS, ACTG1, EHD3, TUBA1B, RAP1A, HINT2, EHD4, PCCB, SUCLG2, VDAC2, COQ8A, RAB5A, FARSB	3.1E-05
GO:0042776 ~ proton motive force-driven mitochondrial ATP synthesis	NDUFA9, NDUFA12, NDUFA2, NDUFS2, SDHA	1.7E-03
GO:0005743 ~ mitochondrial inner membrane	NDUFA9, TST, NDUFA12, NDUFA2, NDUFS2, VDAC2, SLC25A10, SDHA, PHB2	6.9E-04
GO:0016491 ~ oxidoreductase activity	SELENBP1, GPX3, AKR1A1, NDUFS2, AKR1B1, SDHA, PRDX6, IDH3A	1.7E-02
GO:0072659 ~ protein localization to plasma membrane	EHD3, NHERF2, RAP1A, EHD4, SCP2, FLNA	5.6E-03
**Slower FSR in mammary gland of HF mice**		
GO:1990904 ~ ribonucleoprotein complex	RPL4, RPL5, DDX5, RPL30, RPL11, RPL10A, RPL9, RPL6, RPS4X, PCBP2, RPL38, DDX17, HSPA8, NPM1, NOP58, RPS7, RPS8, PABPC4, RPS6, RPL13A, RPSA, RPL23A, ACTN4, EEF2, HNRNPAB, SRP9, ACTA2, HNRNPL, HNRNPK, EPRS1, HNRNPH1, PABPC1, FAU, GAPDH, RPS23	2.1E-20
GO:0051287 ~ NAD binding	LDHB, HADHA, LDHA, CYB5R3, MDH1, IDH2, GPD1, SORD, GAPDH, DLD, NDUFV1, ALDH9A1	3.0E-10
GO:0051082 ~ unfolded protein binding	CCT3, CCT2, HSPA8, NPM1, NUDC, HSP90AA1, HSP90AB1, HSPA5, CLU, HSP90B1, CCT6A, CCT8, CCT7, UGGT1	3.6E-10
GO:0070469 ~ respirasome	NDUFA8, NDUFA13, UQCRB, UQCRC1, NDUFC2, CYCS, MT-CO2, NDUFS1, CYC1, NDUFV2, NDUFV1	9.9E-09
GO:0006631 ~ fatty acid metabolic process	HADHB, OLAH, HADHA, LYPLA1, ACADL, ACSL1, FASN, ACOT1, ACSL4, ACACB, ACACA, SLC27A4	4.6E-04
GO:0008610 ~ lipid biosynthetic process	ACLY, OLAH, ACSL1, FASN, ACSL4, ACACA	4.6E-04
GO:0006099 ~ tricarboxylic acid cycle	ACLY, MDH1, IDH2, ACO1, ACO2, DLAT, FH1	1.6E-04
GO:0006096 ~ glycolytic process	PFKL, PKM, ENO1B, PGK1, ALDOA, GAPDH, HK1	7.0E-04
GO:0000502 ~ proteasome complex	USP14, PSMD6, PSMD11, PSMD14, PSMB2, PSMB3, PSMD2, PSMC1	3.0E-05
GO:0016874 ~ ligase activity	AARS1, ACSL1, MARS1, ACSL4, ACACB, ACACA, ASS1, SARS1, QARS1, NARS1, EPRS1, GLUL, SLC27A4	3.5E-07
KW-0648 ~ Protein biosynthesis	EIF5A, EIF4A1, AARS1, MARS1, EEF2, EEF1A1, SARS1, QARS1, NARS1, EPRS1, EIF3I, EIF2S3X, EIF3D, EIF4E	1.6E-06
GO:0005777 ~ peroxisome	FIS1, PRDX5, MTARC2, ACSL1, IDH2, PLAAT3, ACSL4, VIM, TKT	6.6E-04
GO:0051131 ~ chaperone-mediated protein complex assembly	CCT2, HSP90AA1, HSP90AB1, CLU	2.7E-02
GO:0045454 ~ cell redox homeostasis	TXN1, ERO1A, PRDX5, NNT, PRDX1	1.2E-02
GO:0016192 ~ vesicle-mediated transport	RAB2A, RTN3, ARF1, RAB1B, CLTC, USO1, GDI2, AP2A2, TBC1D4, RAB18, PREB, FOLR1, SEC23B	1.3E-03
GO:0042744 ~ hydrogen peroxide catabolic process	PRDX5, PRDX1, HBB-B2, HBB-B1, APOA4	9.7E-03
GO:0005753 ~ mitochondrial proton-transporting ATP synthase complex	ATP5F1B, ATP5PB, ATP5PO, ATP5MK, ATP5ME	3.0E-04

T-test analysis comparing FSR of proteins in the mammary gland from HF and CON groups found 356 with different rates (raw P-value < 0.05), 72 proteins had a faster FSR and 284 (79.8%) had a slower FSR in mammary glands of mice on a HF diet. Proteins with a faster FSR were large and small subunits of ribosomes, components of complex I and II of the electron transport chain (NDUFA9, NDUFA12, NDUFA2, NDUFS2, SDHA) and PCCB. Proteins with a slower FSR in mammary glands of HF mice functioned in lipid biosynthesis, as citrate cycle enzymes, as components of proteasomes, in vesicle mediated transport and electron transport chain complex V, as well as multiple milk proteins (CSN2, CSN3, CSN1S2A, CSN1S2B, MFGE8) and the transcription factor STAT5A (Table 5 and Fig 5).

### Analysis of BHB and mTOR in liver

**Fig 6:**
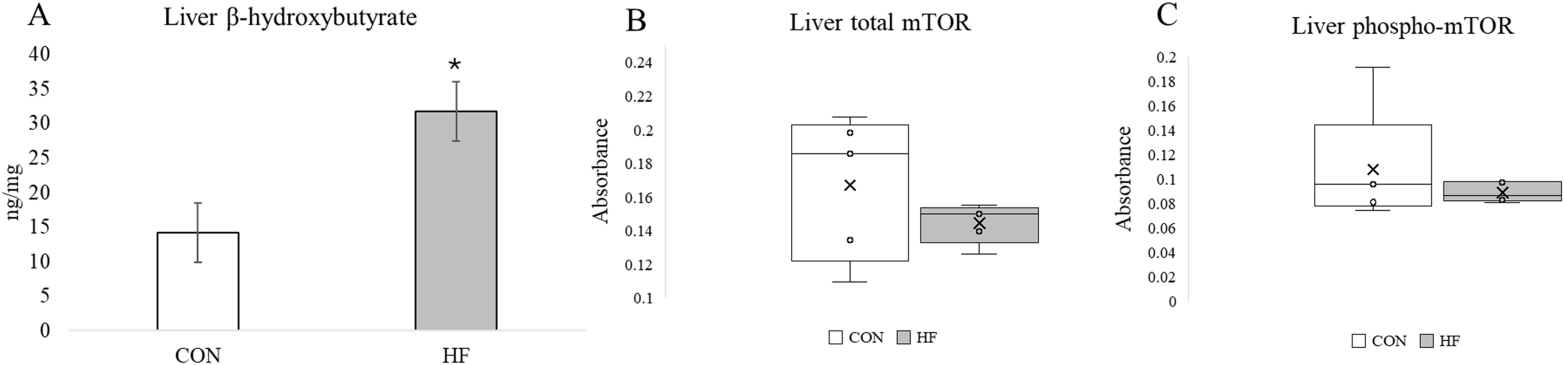
Analysis ofβ-hydroxybutyrate (A), total mTOR (B), and phospho-mTOR (C) in liver of mice fed a high fat (HF) or control (CON) diet.Asterisk represents P < 0.05.

Previous studies demonstrated that suppressing mTOR activity decreased global protein synthetic rates [43–44], and that ketogenic diets inhibit mTOR mediated signaling [45–46]. Our global proteome analysis found mTOR levels significantly lower in liver of HF mice, and the abundance of enzymes (HMGCL, HMGCS2, BDH1) that catalyze reactions for formation of ketone bodies significantly greater in liver of HF diet mice. Analysis of the ketone body β-hydroxybutyrate (BHB) in liver demonstrated mice fed a HF diet had more than double the BHB concentration (31.8 ng/mg) than CON mice (14.2 ng/mg; P = 0.02; Fig 6), supporting the ketogenic effects of the HF diet. Analysis of total mTOR and phosphorylated (activated) mTOR of liver found no difference between HF and CON for total mTOR (P = 0.29; Fig 6) nor phospho-mTOR (P = 0.41; Fig 6), although data trended towards HF having lower abundance of mTOR compared to CON. The mTOR response of CON animals was highly variable likely driving the lack of difference between the two groups.

**Fig 7:**
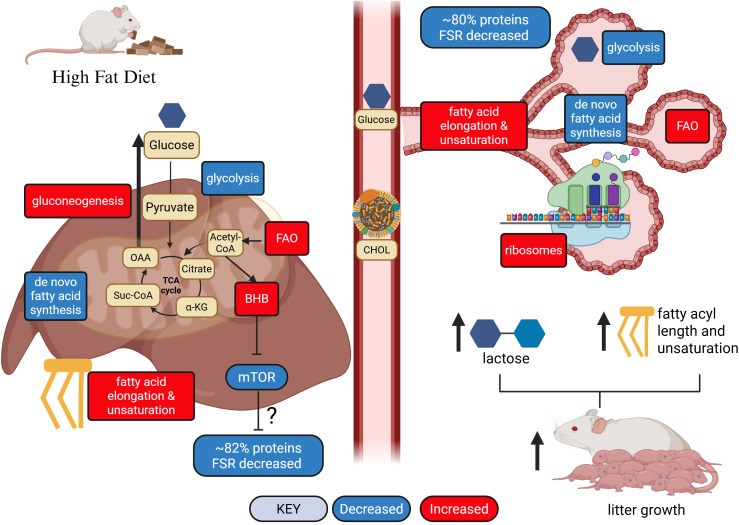
Summary of responses in liver and mammary gland of mice fed a high fat (HF) or control (CON) diet. In the liver, proteins related to fatty acid oxidation (FAO), gluconeogenesis, fatty acid elongation and unsaturation were increased, whereas proteins related to glycolysis and de novo fatty acid synthesis were reduced. In liver, mTOR abundance was reduced, which may be a reason for reduction of fractional synthetic rate (FSR) of ~82% of proteins, with the potential mechanism represented by a question mark. In the mammary gland, proteins related to FAO and fatty acid elongation and unsaturation as well as ribosomal proteins were increased, whereas proteins related to glycolysis and de novo fatty acid synthesis were reduced. Additionally, ~ 80% of protein FSR was decreased in the mammary gland. Mice that received an HF diet had greater milk lactose and fatty acyl chain length and unsaturation likely leading to greater litter growth than CON mice. Reprinted from Biorender.com under a CC BY license, with permission from Biorender.com, original copyright 2026.

A schematic was developed to summarize the overall effects of HF diet on metabolic pathways in the liver and mammary gland revealed by the global proteome analysis described above (Fig 7). Feeding HF diet to mice alters nutrient utilization and metabolism in both the liver and mammary gland by causing a shift towards fatty acid oxidation from glycolysis as the primary means of generating acetyl CoA to form citrate for energy production in cells (Fig 7). In the mammary gland, the shift from glycolysis likely spared glucose for lactose synthesis as indicated by higher milk lactose concentrations [25]. High fat diet also increased fatty acid elongation and unsaturation and reduced *de novo* fatty acid synthesis in both tissues, which translated to greater fatty acyl length and unsaturation in milk [25]. The response of greater fatty acyl length and unsaturation in milk combined with increased lactose concentration in milk as well as seemingly higher protein production capacity with more translational machinery likely contributed to greater pup weight gain in litters of HF dams [24]. In addition, in response to HF, BHB concentrations increased reflecting ketogenic effects of HF diet. Increased levels of BHB inhibit mTOR, and we posit this led to reduced FSR of ~82% of proteins with significantly different FSR in liver (Fig 7).

## Discussion

Comparative analysis of liver and mammary gland global proteomes of peak lactation mice found that HF diet profoundly affected the abundance and FSR of proteins in both organs. The changes in abundance of proteins reflected less dependence on glycolysis and a greater dependence on fatty acid oxidation for energy production in response to HF diet in both the liver and mammary gland. In both tissues, mice on the HF diet showed decreased abundance in pentose phosphate pathway enzymes, which function to produce NADPH, the primary fuel for fatty acid synthesis, and a concomitant decrease in enzymes that mediate *de novo* fatty acid synthesis. Differential FSR analysis found HF diet primarily slowed FSR of proteins, with ~82% and ~80% of proteins slower in the liver and mammary gland of HF fed mice, respectively. Slower FSR was accompanied by a decrease in abundance of multiple proteins including several glycolytic enzymes. However, multiple proteins in both tissues had slower FSR, but increased abundance in mice on HF diet. Although protein degradation was not directly measured, the finding of slower FSR with increased abundance suggests that degradation rate of these proteins was slowed to an even greater extent, which may be interpreted as an increase in a protein’s half-life [47]. Proteins with slower FSR and greater abundance in response to HF diet functioned as citrate cycle enzymes and in the degradation of fatty acids. In the liver, several proteins were also found to have a faster FSR and lower abundance, such as multiple pentose phosphate pathway and glycolytic/gluconeogenic enzymes, and FASN, suggesting an increase in turnover rate of these proteins in response to HF diet. Herein, we discuss the implications of findings regarding maternal metabolism and milk production during lactation. We believe that these data help to expand the general understanding of the impact of HF diet on metabolic pathways and proteostatic processes, which gives potential insight into its effects on health.

Metabolic rate of a tissue relates to protein turnover rate, with highly metabolic tissues having higher protein turnover rates [48]. The liver and mammary glands of lactating mice have very high metabolic rates, and the relatively high number of proteins with FSR estimates between 0.06 and 2 captured in the 24 h labeling period likely reflects the metabolic rate of these organs. Half-lives of proteins range from a few minutes to several days [48], with the relative ratio of synthesis and degradation rates determining protein abundance at equilibrium. We found for liver and mammary of lactating mice, protein FSR showed an overall small, negative correlation with protein abundance, which is similar to findings of others [48]. Turnover rates of proteins vary by organ, but within tissues, proteins with similar functions have similar turnover rates and abundance [47–48]. Like findings from the analysis of brain and liver of mice [48], we found mitochondrial proteins to be among those with the slowest FSR in the liver and mammary gland of lactating mice. This response was reflected in the enrichment of citrate cycle and oxidative phosphorylation proteins and pathways in slowest third for FSR (0.99 FSR). Mitochondrial proteins, including citrate cycle and oxidative phosphorylation enzymes, were also among the most abundant in the liver and mammary glands of mice, again indicating a relatively slower turnover/longer half-life of these proteins. Consistent with our findings that proteins secreted from the mammary gland and liver had the fastest FSR (SERPIN proteins, apolipoproteins, milk proteins and a complement factor), secreted liver proteins (apolipoprotein, chylomicron, complement factors) were previously found to have the fastest rates of turnover in mice [47].

### Reduction of mTOR abundance driven by ketogenic effects of HF diet

Proteome analysis of mTOR abundance via LC-MS/MS found it significantly reduced in liver of HF mice, and analysis for total mTOR and phospho-mTOR via ELISA assay indicated a numeric reduction of both. The high variation in mTOR abundance in the liver of the set of CON mice used for the ELISA validating assay reflected the high variation of this group related to differential metabolic phenotypes (e.g., some CON mice had high BMI). Although the ELISA validating assay did not statistically support a difference between CON and HF, we believe further experiments are warranted to explore the hypothesis that the overall slower liver protein FSR in response to HF diet may be due to a reduced activity related to mTOR abundance. This hypothesis is based on knowledge that mTOR activity suppresses protein degradation while promoting protein synthesis [49]. Increased mTORC1 activity reduces the stability of synthesized polypeptides, whereas suppression of mTORC1 signaling decreased protein synthesis but increased the stability of synthesized polypeptides [43–44]. Caloric restriction associated with increased longevity reduces mTOR activity and in turn absolute synthetic rate of proteins [47]. Ketogenic diets also inhibit mTOR mediated signaling [45–46]. The experimental HF diet used in these studies was ketogenic compared to the CON diet as demonstrated by higher levels of the ketone body, BHB, in the liver of HF mice. Changes in metabolism induced by ketogenic diets parallel alterations that fasting and caloric restriction induce, including increased ketone levels. Thus, we believe it is plausible that the lower mTOR abundance and corresponding overall reduction in FSR in the liver may be related to the ketogenic effects of the HF diet.

### Alterations in proteins related to glycolysis-gluconeogenesis and citrate cycle due to HF diet

Alterations in protein abundance and FSR support that divergent sources of energy (fats versus carbohydrates) between HF and CON diets impacted metabolic pathways needed to produce cellular energy in both the liver and mammary gland. The citrate cycle functions as the central hub for energy production of the cell by oxidizing acetyl CoA [50]. Acetyl CoA is produced primarily from the breakdown of carbohydrates through glycolysis, fatty acids through β-oxidation, and non-essential amino acids. Many of the enzymes that catalyze glycolytic reactions were decreased in abundance in both the liver and the mammary gland of HF diet mice. These decreases in abundance of multiple glycolytic enzymes were accompanied by a decrease in their FSR, reflecting a lower dependence on glucose and other carbohydrates to provide acetyl CoA in HF mice. In contrast, the greater abundance of mitochondrial fatty acid degradation enzymes in HF mice (e.g.,HADHA, HADHB and ACAA2) reflect greater dependence on β-oxidation of fatty acids to produce acetyl CoA.

Citrate cycle intermediates can leave the cycle via cataplerotic reactions to serve as substrates for biosynthesis of glucose, fatty acids and non-essential amino acids [51]. Major cataplerotic enzymes include PCK1 and ATP citrate lyase (ACLY), which pull OAA and citrate from mitochondrial pools for gluconeogenesis and lipogenesis, respectively [51]. In both the liver and the mammary gland, the abundance of ACLY was reduced, reflecting a lower need for *de novo* fatty acid synthesis. Abundance of PCK1 was greater in the liver of HF mice. PCK1, the first rate-limiting enzyme of gluconeogenesis, catalyzes the conversion of OAA into PEP [50], and is critical for maintaining both glucose and lipid homeostasis [52]. For continued cycling of the citrate cycle, intermediates must also be replaced through anaplerotic reactions. Pyruvate carboxylase (PCX) serves as a major anaplerotic enzyme, catalyzing the carboxylation of pyruvate into OAA [51]. Combined, the greater abundance of PCX, glutamic-oxaloacetate transaminase 2 (GOT2), and PCK1 in the liver of HF mice, support the potential for greater gluconeogenic capacity. Additionally, in the liver, the decrease in abundance of the glycolytic/gluconeogenic enzymes ALDOB and FBP1 was accompanied by a faster FSR in HF mice, indicating a higher turnover rate of these proteins. Fructose-1,6-bisphosphatase (FBP1) is rate-limiting to gluconeogenesis, and so changes in FSR, and potentially degradation, may enable a more rapid response to glucose demands. Although changes in protein abundance does not necessarily reflect changes in its activity, we interpret alterations in the abundance of these enzymes as an increase in gluconeogenic capacity of liver of HF diet mice. An increase in hepatic gluconeogenic capacity, combined with more glycerol available from TG catabolism, likely increased the availability of substrates for lactose synthesis in the mammary gland, and thus may underlie the higher lactose content of milk of HF diet mice [21,25].

Acetyl CoA allosterically regulates PCX activity. The high levels of acetyl CoA produced by fatty acid oxidation inhibit pyruvate dehydrogenase activity, while promoting the PCX mediated reaction [50]. The lower abundance of multiple components of pyruvate dehydrogenase enzyme in the liver may partly reflect this inhibition. Potentially higher levels of acetyl CoA from increased β-oxidation, combined with decreased abundance of citrate synthase in the liver, shunted excess acetyl CoA toward ketone body production in the liver of HF mice, as reflected in the greater abundance of the ketogenic enzymes HMGCL, HMGCS2, and BDH1 and increased liver BHB concentration.

Branched chain fatty acids and branched chain amino acids enter the citrate cycle as succinyl CoA following conversion to propionyl-CoA through a series of anaplerotic reactions catalyzed in part by dual subunit propionyl-CoA carboxylase (PCC) [50]. Alterations in proteomes of both organs reflect a potential increase of anaplerotic supply of intermediates into the citrate cycle through the PCC pathway. In the liver, the FSR of PCCB was faster in HF mice. In the mammary gland, HF diet increased PCCB FSR as well as the abundance of its alpha subunit (PCCA). In the mammary gland, the abundance of two enzymes that function to catabolize branch chain amino acids, branched chain amino acid amino transferase (BCAT2) and branched chain keto acid dehydrogenase (BCKDHB), were more abundant in HF mice. These findings support the use of both branched chain amino acids and odd chain fatty acid sourced carbons to replenish citrate cycle intermediates in the mammary gland.

High fat diet alteration in abundance of enzymes that control cataplerotic and anaplerotic reactions, like PCX and PCK1, likely affected the flow of carbons through citrate cycle intermediates in both the mammary gland and liver. Altering PCX abundance of a normal kidney cell altered the flow of carbons through the citrate cycle [53]. Greater PCX abundance increased the flux of carbon from labeled pyruvate into all citrate acid cycle intermediates, increased ketone production, increased abundance of mitochondrial fatty acid transporters, the fatty acid β-oxidation enzyme, HADHB, and the abundance of PCCB, GOT2 and PCK2 [53]. Thus, the greater PCX abundance in liver in response to HF diet possibly increased the carbon flow through citrate cycle intermediates.

The HF diet fed to the mice was a mix of saturated and unsaturated fatty acids being a combination of lard that primarily consists of palmitic acid and oleic acid [54], and soybean oil, which is enriched with unsaturated fatty acids including linoleic acid and oleic acid [54]. Treatment of kidney cells with equal proportions of the unsaturated fatty acid, α-linolenic acid, and the saturated fatty acid, palmitate, decreased ACLY while increasing the abundance of multiple electron transport chain proteins in complex III, IV and V [53]. These alterations in protein abundance were similar to the response of liver to the HF diet. In addition, fatty acids regulate PCX and PCK1, therefore, a combination of factors are likely regulating the potential for HF diet to influence citrate cycle carbon flux.

In the mammary gland, HF diet increased the abundance and reduced FSR of three citric acid cycle enzymes aconitase (ACO2), isocitrate dehydrogenase 2 (IDH2) and succinate-CoA ligase GDP-forming subunit beta (SUCGL2) and increased FSR of IDH3 and succinate dehydrogenase alpha subunit (SDHA). Feeding HF diet to mice also increased the abundance of multiple proteins in complex I of the electron transport chain. These changes likely facilitated greater energy production for greater milk synthesis, reflecting the higher rate of growth of pups in litters of HF dams [24,25]. The greater abundance of 15 ribosomal protein as well as multiple protein transporters in the mammary gland of HF mice also likely increased milk production capacity.

### Alterations in proteins related to lipid metabolism due to HF diet

Our previous lipidome analysis of the plasma, mammary gland, and milk of mice fed HF diet found longer total carbon length and number of unsaturated bonds across the three fatty acyl groups of TG [25]. This altered distribution of TG in plasma may be partly due to the increased abundance of 3-hydroxyacyl-CoA dehydratase 3 (HACD3), peroxisomal trans-2-enoyl-CoA reductase (PECR) and acyl-CoA synthetase long chain family member 1 (ACSL1) in the liver of HF mice. HACD3 and PECR catalyze elongation reaction cycles in the synthesis of very long chain fatty acids, and ACSL1 catalyzes the conversion of long chain fatty acids to their active form as acyl-CoAs. Longer chains and greater unsaturation of mammary and milk lipids in HF animals may be due to both their uptake from circulation and their greater synthesis in the mammary gland. Hydroxysteroid 17-beta dehydrogenase 12 (HSD17B12) and fatty acid desaturase 1, 2 and 3 (FADS1, FADS2, and FADS3) were more abundant in the mammary glands of HF diet fed mice. HSD17B12 catalyzes the synthesis of long and very long chain fatty acids, whereas the FADS enzymes function in the biosynthesis of highly unsaturated fatty acids from the essential polyunsaturated fatty acids, linoleic acid (18:2n-6) and α-linolenic acid (18:3n-3 cis), which are included in moderate proportions in the HF diet.

The lower proportion of TG with shorter chain fatty acids and fewer unsaturated bonds in plasma, mammary glands, and milk of HF diet mice can also be traced to changes in liver and mammary proteomes. Feeding HF diet decreased the abundance of FASN in both the liver and mammary gland. FASN catalyzes *de novo* synthesis of long chain saturated fatty acids, and HF diet reduced the abundance of several critical *de novo* fatty acid synthesis enzymes, ACLY, acetyl CoA carboxylase (ACACA), and acyl-CoA synthetase short chain family member 2 (ACSS2). In the mammary gland, FSR of ACLY and ACACA was slower, with no change in abundance, suggesting a slower turnover rate of these proteins. Moreover, in both tissues, the abundance of stearoyl-CoA desaturases (SCD1,2,3) was reduced. SCD play a key role in mediating *de novo* fatty acid synthesis, and catalyze the mono-unsaturation of long chain fatty acids [55]. In our manuscript that describes HF diet alterations in liver, plasma, mammary and milk lipid profiles, we discuss the potential consequences of maternal HF diet changes in TG content of milk on the developing neonate including increased exposure to inflammatory lipids, lower digestibility of very long chain fatty acids versus short and medium chain fatty acids, and long-term increased risk of developing obesity [25].

There were increased and decreased abundance of peroxisomal proteins in the liver. Proteins increased in abundance mediated transport of fatty acids into the peroxisome (ABCD2, ACSL1, SCL27A2), functioned in fatty acid oxidation and in detoxification reactions. HF increased abundance of two enzymes that catalyze fatty acid oxidation, enoyl-CoA delta isomerase 2 (ECI) and enoyl coenzyme A hydratase 1 (ECH1), with increased abundance preventative of hepatic steatosis [56]. Proteins that function in detoxification reactions include AGXT, which functions to detoxify glyoxylate [57], LONP2, which functions to maintain peroxisome homeostasis by degrading oxidized proteins, and DHRS4, which reduces steroid and xenobiotic compounds [58]. HF diets also decreased abundance of EHHADH, which catalyzes peroxisomal β-oxidation reactions and PEX11A, which regulates peroxisomal biogenesis. Changes in peroxisomal proteins in response to HF diet likely reflect the role of peroxisomes in mediating lipid homeostasis [59]. Our previous analysis found plasma triglycerides were not different between lactating CON and HF mice, moreover liver lipid content was lower in HF mice [24] suggesting that lipid homeostasis was maintained in HF mice. The decreased abundance of SCD1 and related SCD proteins may also be hepatoprotective [60].

High fat diet increased the abundance of multiple liver proteins involved in xenobiotic metabolism including cytochrome P450 (CYP) proteins and uridine 5’-diphospho-glucuronosyltransferase (UGT). CYP and UGT isoforms also possess roles in lipid metabolism. UGT aids in the elimination of fatty acids through glucuronidation to enhance elimination through excretion [61]. Cytochrome p450 proteins primarily target polyunsaturated fatty acids to produce oxylipins, like epoxides, which can have anti-inflammatory type properties [62]. Thus, the lack of evidence of hepatic steatosis or dyslipidemia in mice response to HF diet [24] may be partly due to the increased hepatic detoxifying response to the high lipid load.

### Limitations

There are several limitations to this study and interpretations of data. Among the limitations includes that a change in protein abundance does not necessarily reflect a change in protein activity. Further studies need to be conducted to confirm interpretations of changes in protein abundance affecting the metabolic pathways highlighted above. Moreover, although rodents are commonly used to study physiological and nutritional responses in health and disease states, these data are not necessarily directly translatable to human systems. Mice used in our study were fed a HF diet prior to breeding to establish a model of pre-pregnancy obesity. Mice remained on the HF diet through breeding and throughout the entire study, but did not demonstrate divergent weight and body mass index between CON and HF groups to peak lactation [24]. Our analysis of feed intake and feeding behavior throughout gestation and lactation in these animals demonstrated increases in hyperphagia in both CON and HF animals as lactation progressed, but HF mice spent less time consuming the calorically dense diet and more time in the nest [63]. Thus, changes in maternal behavior and milk composition associated with HF diet together may contribute to greater growth rate of their litters. Additionally, more time in the nest, rather than eating, resulted in similar caloric intake between CON and HF during peak lactation, which likely contributed to loss of HF weight and BMI to match CON at the end of the study [24,63]. In addition to increased food intake, rodents mobilize 60–70% of their fat stores to support lactation [64–65]. Women also experience lipolysis to support lactation [64–65], but the state of maternal obesity is most often maintained through gestation and lactation [66]. Therefore, although these data provide an understanding of how the global proteome is altered in the liver and mammary gland in response to HF diet fed throughout reproductive stages in a rodent, translation of findings to humans, and the consequence to their offspring, need to be made in the context of women’s physiology and diet.

## Conclusions

High fat diet decreased the FSR of the majority of proteins identified as significantly different in the liver and mammary gland of peak lactation mice. Although further studies are needed, we postulate that the decrease in mTOR abundance in liver of mice on HF diet may underscore the overall slower protein FSR, as ketogenic diets decrease mTOR activity, and decreased mTOR activity decreases protein synthesis. Decreased abundance of glycolytic enzymes and increased abundance of enzymes that mediate fatty acid oxidation in HF mice reflected use of fatty acids rather than carbohydrates to fuel cellular energy needs in both tissues. The abundance of enzymes that mediate *de novo* fatty acid synthesis and mono-unsaturation was decreased and enzymes that catalyzed the elongation and desaturation of polyunsaturated fatty acids were increased with HF diet in both tissues. These alterations underlie the differences in lipid profiles of mice on HF diet compared to CON diet. Increased abundance of gluconeogenic enzymes in the liver and reduced glycolytic enzymes, potentially reflects a greater gluconeogenic capacity of HF dams. Although enzyme activity was not measured, we posit that the potential for increased availability of glucose in HF dams supported greater lactose synthesis in the mammary gland, reflecting the higher lactose content in milk of mice on HF diet. High fat diet also increased the abundance of ribosomal proteins and protein transporters in the mammary gland, potentially increasing milk production capacity. Alterations in global proteome and FSR of liver and mammary gland enable a greater understanding of changes in milk composition in response to HF diet that likely underscore the greater growth rate of their litters. Findings also expand the general understanding of HF diet impacts on metabolic pathways and proteostatic processes.

## Supporting information

S1 Text Supplemental Tables and Figures. Supplemental Tables and Figures were uploaded to the Purdue University Research Repository and are freely available. Supplemental Tables and Figures can be accessed using this citation and DOI: Beckett, L.M; Lichti, N. I.; Casey, T.M. (2026). The impact of high fat diet on global protein abundance and fractional synthetic rate in liver and mammary gland of peak lactation ICR mice. (Version 2.0). Purdue University Research Repository. doi:10.4231/XX3A-7918.
(DOCX)

S2 Text Appendix A. Definitions of all protein abbreviations in Fig 5A.
(XLSX)

S3 Text Appendix B. Definitions of all protein abbreviations in Fig 5B.
(XLSX)

## Abbreviation:

BHB: β-Hydroxybutyrate
CON: control
D_2_O: deuterium oxide
DAP: differentially abundant proteins
EDC: N-(3-Dimethylaminopropyl)-N′-ethylcarbodiimide hydrochloride
ESI: electrospray ionization
FSR: fractional synthetic rate
GC-MS: gas chromatography mass spectrometry
HF: high fat
HPLC: high performance liquid chromatography
LC-MS: liquid chromatography mass spectrometry
LC-MS/MS: liquid chromatography tandem mass spectrometry
LFQ: label free quantification
MIDA: mass isotopomer distribution analysis
mTOR: mammalian target of rapamycin
OAA: oxaloacetate
PCA: principal component analysis
PEP: phosphoenolpyruvate
RT: room temperature
TG: triacylglycerol
