# Supplementary material for: The impact of high fat diet on global protein abundance and fractional synthetic rate in liver and mammary gland of peak lactation ICR mice
Source: PLoS One. 2026 Apr 10;21(4):e0346148. doi: 10.1371/journal.pone.0346148 (PMC13068346; doi:10.1371/journal.pone.0346148)
Supplement: S1 Text — Supplemental Tables and Figures were uploaded to the Purdue University Research Repository and are freely available. Supplemental Tables and Figures can be accessed using this citation and DOI: Beckett, L.M; Lichti, N. I.; Casey, T.M. (2026). The impact of high fat diet on global protein abundance and fractional synthetic rate in liver and mammary gland of peak lactation ICR mice. (Version 2.0). Purdue University Research Repository. doi:10.4231/XX3A-7918. (DOCX) [file pone.0346148.s001.docx]

Supplemental Tables and Figures were uploaded to the Purdue University Research Repository and are freely available. Supplemental Tables and Figures can be accessed using this citation and DOI: Beckett, L.M; Lichti, N. I.; Casey, T.M. (2026). The impact of high fat diet on global protein abundance and fractional synthetic rate in liver and mammary gland of peak lactation ICR mice. (Version 2.0). Purdue University Research Repository. [doi:10.4231/XX3A-7918](https://doi.org/10.4231/XX3A-7918)
